# Novel rRNA transcriptional activity of NhaR revealed by its growth recovery for the *bipA*-deleted *Escherichia coli* at low temperature

**DOI:** 10.3389/fmolb.2023.1175889

**Published:** 2023-04-20

**Authors:** Eunsil Choi, Ahhyun Huh, Jihwan Hwang

**Affiliations:** ^1^ Department of Microbiology, Pusan National University, Busan, Republic of Korea; ^2^ Microbiological Resource Research Institute, Pusan National University, Busan, Republic of Korea

**Keywords:** NhaR, BipA, GTPase, capsule, biofilm, ribosome, rRNA

## Abstract

The BipA protein is a universally conserved GTPase in bacterial species and is structurally similar to translational GTPases. Despite its wide distribution, BipA is dispensable for growth under optimal growth conditions but is required under stress conditions. In particular, *bipA*-deleted cells (ESC19) have been shown to display a variety of phenotypic changes in ribosome assembly, capsule production, lipopolysaccharide (LPS) synthesis, biofilm formation, and motility at low temperature, suggesting its global regulatory roles in cold adaptation. Here, through genomic library screening, we found a suppressor clone containing *nhaR*, which encodes a Na^+^-responsive LysR-type transcriptional regulator and whose gene product partially restored the growth of strain ESC19 at 20°C. The suppressed cells showed slightly reduced capsule production and improved biofilm-forming ability at 20°C, whereas the defects in the LPS core and swimming motility were not restored but aggravated by overexpression of *nhaR*. Notably, the overexpression partially alleviated the defects in 50S ribosomal subunit assembly and rRNA processing of ESC19 cells by enhancing the overall transcription of rRNA. Electrophoretic mobility shift assay revealed the association of NhaR with the promoter of seven *rrn* operons, suggesting that NhaR directly regulates rRNA transcription in ESC19 at 20°C. The suppressive effects of NhaR on ribosomes, capsules, and LPS were dependent on its DNA-binding activity, implying that NhaR might be a transcriptional factor involved in regulating these genes at 20°C. Furthermore, we found that BipA may be involved in adaptation to salt stress, designating BipA as a global stress-responsive regulator, as the deletion of *bipA* led to growth defects at 37°C and high Na^+^ concentrations without ribosomal defects.

## 1 Introduction

Bacterial cells undergo several physiological and physical changes to adapt and survive under unfavorable conditions, when exposed to stressful environments, including extreme temperatures, pH shifts, limited nutrients, oxidative stress, osmotic stress, and antimicrobial agents. These changes include the regulation of growth rate, protein synthesis, cell morphology, and membrane synthesis. To support these protective mechanisms, expressions of numerous stress-responsive genes are up- and downregulated at the transcription, translation, or post-translational levels.

Ribosomes are the machineries for protein synthesis and play a significant role in determining the overall gene expression profile of the cell. Therefore, cells must regulate the appropriate intracellular level of ribosomes and translation rate under stressful conditions. Numerous protein factors are associated with ribosomes, which regulate ribosome assembly and protein synthesis in response to environmental stresses (Starosta et al., 2014). For instance, under nutrient starvation conditions, the 50S ribosomal subunit assembly factor ObgE binds to a stringent response alarmone (p)ppGpp instead of GTP (Buglino et al., 2002; Jiang et al., 2006; Jiang et al., 2007). This association enhances its binding affinity to the 50S ribosomal subunit, resulting in the dissociation of the 70S ribosome into subunits (Feng et al., 2014). At high intracellular Mg^2+^ concentrations, ribosomes undergo conformational changes obstructing translational activity, and LepA rescues ribosomes stalled on mRNA, consequently allowing translation to resume (Pech et al., 2011).

Among the stress-responsive ribosome-associated factors, BipA is highly conserved in bacteria, is also found in chloroplasts (Margus et al., 2007; Wang et al., 2008), and is required for growth under a wide range of stressful conditions, such as cold, acidic, oxidative, and detergent stresses, and the presence of antibiotics or antimicrobial peptides (Farris et al., 1998; Pfennig and Flower, 2001; Kiss et al., 2004; Wang et al., 2008; Neidig et al., 2013; Crane et al., 2021). BipA exhibits high sequence and structural homology with translational GTPases (trGTPases), such as EF-G and LepA, and shares binding sites on the ribosome (Kumar et al., 2015; Ero et al., 2016). However, unlike these trGTPases, which regulate critical checkpoint steps during translational initiation, elongation, and termination or translational fidelity (Shoji et al., 2010; Maracci and Rodnina, 2016), BipA has not been reported to be involved in any of those translation processes; instead, it participates in ribosome assembly at low temperature by promoting the integration of ribosomal protein (r-protein) L6 into the 50S ribosomal subunit precursor (Choi and Hwang, 2018).

Upon exposure to ultraviolet (UV) radiation, desiccation, antibiotics, antimicrobial peptides, extreme pH, temperature, salt, or pressure, bacterial cells produce capsular polysaccharides or biofilms as a physical barrier to protect cells and assist survival under hostile conditions (Slack and Nichols, 1981; Dunne et al., 1993; Ophir and Gutnick, 1994; Campos et al., 2004; Dubois et al., 2019; Yin et al., 2019). Lipopolysaccharides (LPS), composed of lipid A, core oligosaccharide, and O-antigen, are the main component of the outer leaflet of the outer membrane and contribute to membrane integrity and permeability (Yethon et al., 1998). Upon exposure to cold stress, bacteria synthesize LPS with short or unsaturated acyl chains to maintain membrane integrity and fluidity (Vorachek-Warren et al., 2002; Herrera et al., 2021). *Escherichia coli* cells with defective LPS allow for easier penetration of harmful compounds such as chlorhexidine and quaternary ammonium, eventually damaging the bacterial membrane and resulting in the leakage of intracellular components (Russell and Furr, 1986; McDonnell and Russell, 1999). Finally, flagella allow bacteria to evade adverse environments to more favorable locations (He and Bauer, 2014). BipA plays a pivotal role in the regulation of the above-mentioned virulence factors, as previously demonstrated by us (Choi et al., 2020a). At 20°C, *bipA*-deleted cells accumulated excessive capsular polysaccharides with concomitant loss of biofilm formation and flagella-mediated motility. In addition, normal LPSs are not synthesized, accumulating the truncated form of the LPS core, and being sensitive to bile salts. Thus, it is assumed that BipA collectively supports growth at low temperature by regulating virulence factors and ribosome assembly.

In our recent reports, genomic library screening was carried out to identify a suppressor that restores the cold sensitivity of the *bipA*-deleted strain (Choi et al., 2020a; Choi et al., 2020b). We demonstrated that *rplT* and *yebC*, which encode r-protein L20 and putative transcriptional regulator YebC, respectively, are suppressor genes of *bipA* deletion. Overexpression of *rplT* significantly recovered the abnormal 50S ribosomal subunit assembly with a diminished accumulation of rRNA precursors in the *bipA*-deleted strain at 20°C (Choi et al., 2020b). Notably, this suppressor could not reverse the defects of *bipA*-deleted cells in surface polysaccharides, such as capsules, LPS, and biofilms. Unlike L20, overexpression of *yebC* suppressed defects in capsule production, LPS core maturation, and biofilm formation at 20°C without restoration of ribosome assembly (Choi et al., 2020a). Our findings suggest that the cold-sensitive phenotype of *bipA* deletion can be attributed to either aberrant ribosomes or defective surface polysaccharides, which place BipA as a global regulatory GTP-binding protein.

In this study, we investigated another positive suppressor of *bipA* deletion, NhaR. NhaR was first identified as a positive transcriptional regulator of *nhaA*, which is located upstream of the same operon and encodes the major Na^+^/H^+^ antiporter NhaA (Rahav-Manor et al., 1992). NhaR is an LysR-type transcriptional regulator (LTTR), the most abundant transcriptional regulator family in bacteria (Henikoff et al., 1988). The LTTR superfamily has a conserved structure, with a DNA-binding domain containing a helix-turn-helix (HTH) motif at the N-terminus and a co-inducer-binding domain at the C-terminus (Maddocks and Oyston, 2008). NhaR binds to Na^+^ as a co-inducer and regulates transcription by binding to the promoter regions of target genes (Rahav-Manor et al., 1992). To date, only three target genes have been identified as transcriptionally upregulated by NhaR: i) *nhaAR* operon, ii) *osmC* encoding an osmotic shock-inducible protein with peroxidase activity, and iii) *pgaABCD* operon involved in the production, modification, and export of poly-β-1,6-N-acetyl-D-glucosamine (PGA), which is a polysaccharide adhesin required for attachment during biofilm formation (Rahav-Manor et al., 1992; Toesca et al., 2001; Goller et al., 2006).

Our efforts to elucidate the role of NhaR in suppression revealed that in addition to *nhaAR*, *osmC*, and *pgaABCD*, NhaR is implicated in the regulation of the capsule, LPS, and motility at low temperature. More importantly, NhaR is likely to function as a trans-acting element in the promoter of ribosomal RNA genes to modulate and adjust the expression of rRNA at low temperature.

## 2 Materials and methods

### 2.1 Bacterial strains and growth conditions

The *E. coli* strains used in the present study are listed in Table 1. The MG1655-originated deletion strain ESC61 (*nhaR*::kan) was constructed by P1 transduction, using P1 lysates prepared from JW0019 (*nhaR*::kan, BW25113) as a donor. The kanamycin cassette in ESC19 (*bipA*::kan) was removed using pCP20 (Datsenko and Wanner, 2000), and the resulting strain was transduced with two different P1 lysates, yielding strains ESC62 (Δ*bipA*, *nhaR*::kan) and ESC69 (Δ*bipA*, *lacZ*::kan). The deletion of genes was confirmed by polymerase chain reaction (PCR) using primer pairs (Supplementary Table S1). The strains were grown in Luria–Bertani (LB) medium (10 g/L tryptone, 5 g/L yeast extract, and 5 g/L NaCl) at 37°C or 20°C. To monitor growth in LB medium, the overnight cultures were diluted 10^2^-fold with fresh medium and grown at 20°C. The optical density of the 5-fold diluted cultures was measured at 600 nm (OD_600_) every 6 h. To test the sensitivity of bacterial growth to NaCl, overnight cultures were diluted 10^2^-fold in LB medium containing 87 mM KCl instead of NaCl (LBK) and incubated at 37°C until the exponential phase. The cultures were diluted 5-fold into LBK medium supplemented with 500 mM NaCl or 300 mM NaCl, and the diluted cultures were incubated at 37°C or 20°C. The cultures were diluted with the same medium and the OD_600_ was measured every 2 h at 37°C or 6 h at 20°C. The pH of the medium with NaCl was adjusted to 8.5 with KOH. For the bile salt sensitivity test, overnight cultures were diluted to OD_600_ of 0.2 in LB medium, followed by further dilution of 10^2^-fold. The diluted sample (3 μL) was streaked onto LB agar plates supplemented with various concentrations of bile salts (48305; Sigma-Aldrich), post which, the plates were incubated at 37°C or 20°C. Ampicillin (Amp; 100 μg/mL), chloramphenicol (Cm; 50 μg/mL), or kanamycin (Kan; 50 μg/mL) was added to the medium as needed.

**TABLE 1:** Bacterial strains and plasmids used in this study.

Strains	Description	Source or References
MG1655	F^−^ λ^−^ *ilvG* ^−^ *rfb*-*50* *rph*-*1*, *Escherichia coli* K-12	Blattner et al. (1997)
ESC19	*bipA*::kan, MG1655	Choi and Hwang (2018)
ESC61	*nhaR*::kan, MG1655	This study
ESC62	Δ*bipA*, *nhaR*::kan, MG1655	This study
ESC69	Δ*bipA*, *lacZ*::kan, MG1655	This study
JW0019	*nhaR*::kan, BW25113	Baba et al. (2006)
**Plasmids**		
pACYC184	Cm^R^, Tc^R^, *ori* p15A, cloning vector	New England Biolabs
pACYC184BipA	*bipA* ^*+*^, pACYC184	Choi and Hwang (2018)
pBIS02-2	*rplT* ^*+*^, pACYC184	Choi et al. (2020b)
pBIS05-2	*yebC* ^*+*^, pACYC184	Choi et al. (2020a)
pBIS07-1	*nhaR* ^+^, pACYC184	This study
pBIS07-1_Q35A_	*nhaR* (Q35A), pACYC184	This study
pBIS07-1_Q40A_	*nhaR* (Q40A), pACYC184	This study
pBIS07-1_Q35AQ40A_	*nhaR* (Q35A, Q40A), pACYC184	This study
pBIS07-2	*insB1* ^+^-*insA* ^*+*^, pACYC184	This study
pRS414	*bla-Tl*_*4*_ *-Eco*RI*-Sma*I*-Bam*HI *lacZ′*	Simons et al. (1987)
pRS414-P*rrnA*	*rrnA* promoter region*-lacZ′*, pRS414	This study
pRS414-P*rrnB*	*rrnB* promoter region*-lacZ′*, pRS414	This study
pRS414-P*rrnC*	*rrnC* promoter region*-lacZ′*, pRS414	This study
pRS414-P*rrnD*	*rrnD* promoter region*-lacZ′*, pRS414	This study
pRS414-P*rrnE*	*rrnE* promoter region*-lacZ′*, pRS414	This study
pRS414-P*rrnG*	*rrnG* promoter region*-lacZ′*, pRS414	This study
pRS414-P*rrnH*	*rrnH* promoter region*-lacZ′*, pRS414	This study
pET28a	N-terminal His_6_-tag expression vector	Novagen
pET28NhaR	*nhaR* ^+^, pET28a	This study
pET28NhaR_Q35AQ40A_	*nhaR* (Q35A, Q40A), pET28a	This study
pET28Fis	*fis* ^+^, pET28a	This study
pCP20	*λcI857* (ts) *repA101* (ts) *oriR101 bla cat λpR-FLP*	Datsenko and Wanner (2000)

*bla*, Ampicillin resistance; kan, Kanamycin resistance; Cm^R^ or *cat*, Chloramphenicol resistance; Tc^R^, tetracycline resistance.

### 2.2 Construction of a genomic library and suppressor screening

A genomic library construction of the *bipA*-deleted strain and screening of suppressors restoring the cold sensitivity of ESC19 were performed as described in a previous study (Choi et al., 2020b). Briefly, the genomic DNA of ESC19 cells was extracted and partially digested with *Sau*3AI, followed by ligation into pACYC184 at the *Bam*HI site. ESC19 cells were transformed using the ESC19 genomic library and grown at 20°C. Colonies larger than ESC19 harboring an empty vector pACYC184 were screened as suppressors of cold sensitivity in the strain ESC19.

### 2.3 Plasmid construction

To construct truncated clones from pBIS07, DNA fragments were amplified by PCR from pBIS07 using the primer pairs listed in Supplementary Table S1. The DNA fragments were digested with *Bam*HI and ligated into the same site of pACYC184, yielding pBIS07-1 and pBIS07-2. Site-directed mutagenesis PCR was performed on pBIS07-1 to construct pBIS07-1_Q35A_, pBIS07-1_Q40A_, and pBIS07-1_Q35AQ40A_. The PCR-amplified DNA fragments of *nhaR* and *fis* were digested with *Nde*I-*Sal*I and *Nde*I-*Hin*dIII, respectively, and then ligated into the same sites of pET28a, yielding pET28NhaR and pET28Fis, respectively. The double mutant *nhaR* DNA was cloned into the pET28a vector, yielding pET28NhaR_Q35AQ40A_. For the β-galactosidase assay, the promoters of seven *rrn* operons were amplified by PCR and digested with *Eco*RI-*Bam*HI. Then, the insert DNAs were cloned into pRS414 to yield pRS414-P*rrnA*, pRS414-P*rrnB*, pRS414-P*rrnC*, pRS414-P*rrnD*, pRS414-P*rrnE*, pRS414-P*rrnG*, and pRS414-P*rrnH*. The plasmids used in this study are listed in Table 1.

### 2.4 Spotting assay

The overnight cultures grown in LB medium at 37°C were diluted to OD_600_ of 0.2 and further diluted 10^1^, 10^2^, 10^3^, 10^4^, and 10^5^-fold. Three microliters of each diluted sample was spotted on LB agar plates, and the plates were incubated at 37°C or 20°C. To examine salt sensitivity, each strain was inoculated into LBK medium without NaCl and cultivated overnight at 37°C. Overnight cultures were diluted in LBK medium. The diluted sample (3 μL) was spotted onto LBK agar plates with or without NaCl, followed by incubation at 37°C or 20°C.

### 2.5 Observation of cell morphology

The overnight cultures grown in LB medium at 37°C were diluted to the OD_600_ of 0.04 in the same medium, followed by a further dilution of 10^3^-fold. The diluted samples (100 μL) were spread on LB agar plates and incubated at 37°C or 20°C. Colony images were acquired using CanoScan LiDE 700F. For macrocolony observation, overnight cultures were diluted to an OD_600_ of 0.02 in the same medium. Three microliters of the diluted cultures were spotted onto LB agar plates and incubated at 37°C or 20°C. The macrocolonies were photographed with a Canon EOS 5D Mark IV camera.

### 2.6 Biofilm assay

Overnight cultures were diluted 10^2^-fold into 2.5 mL of fresh medium in glass tubes and cultivated at 37°C for 24 h or 20°C for 48 h with shaking. After incubation, the cultures were gently removed, and the glass tubes were rinsed with 2.7 mL of 0.85% saline solution, followed by staining with 0.1% crystal violet for 5 min. After washing three times with 3 mL of distilled water, crystal violet was solubilized with 95% ethyl alcohol, and the absorbance of the dissolved crystal violet was measured at 540 nm using a Multiskan^TM^ GO (Thermo Fisher Scientific^TM^) Microplate Spectrophotometer. The absorbance of blank glass tubes at 540 nm was subtracted from each measurement.

### 2.7 Swimming motility assay

From cells growing exponentially at 37°C, 1 mL of cultures per 0.5 of OD_600_ was harvested by centrifugation for 5 min at 900 ×*g*. The supernatants were discarded, and the pellets were resuspended in 100 μL of LB medium by tapping. Five microliters of the suspension were injected into the middle of LB plates containing 0.3% agar (Bacto^TM^ agar, BD Difco™). The plates were then incubated at 37°C or 20°C. Swimming speed (μm/h) was calculated by dividing the average migration distance (μm) by the incubation time (h).

### 2.8 Sucrose density gradient sedimentation

The cells were cultivated at 37°C or 20°C until OD_600_ reached ∼0.5. The cultures were treated with 250 μg/mL Cm for 5 min followed by centrifugation to harvest them. The cell pellets were washed and resuspended in Buffer BP [20 mM Tris-HCl (pH 7.5), 10 mM MgCl_2_, 100 mM NH_4_Cl, and 5 mM β-mercaptoethanol (BME)] for polysome analysis or Buffer BS [20 mM Tris-HCl (pH 7.5), 1 mM MgCl_2_, 100 mM NH_4_Cl, and 5 mM BME] for subunit analysis. The cells were lysed by repeated freeze-thaw cycles, followed by centrifugation for 30 min at 30,000 ×*g* and 4°C to obtain cleared cell lysates. The resulting lysate was loaded onto 10 mL of 5%–40% sucrose gradient prepared in Buffer BP or 5%–25% sucrose gradient prepared in Buffer BS. Centrifugation and fractionation were performed as previously described (Choi et al., 2020b).

### 2.9 Northern blotting

Total RNA was extracted as previously described (Choi et al., 2020b). An equal volume of formamide was added to the RNA samples, and the mixture was heat-treated for 10 min at 72°C to denature the RNA. Four micrograms of RNA each were electrophoresed on 1% agarose gels in 0.5× TBE [45 mM Tris-borate (pH 8.3) and 1 mM ethylenediaminetetraacetic acid (EDTA)] for 30 min at 95 V. After electrophoresis, RNAs were transferred to membranes (Amersham Hybond™-N^+^, GE Healthcare) by capillary transfer and fixed to the membranes by cross-linking the blot with a UV crosslinker. In hybridization buffer [100 mM sodium phosphate (pH 7.2), 0.2 mM EDTA, 1% sodium dodecyl sulfate (SDS), and 1 mg/mL bovine serum albumin (BSA)], the membranes were pre-hybridized for 4 h at 65°C with 50 μg/mL denatured salmon sperm DNA, followed by treatment of 500 ng/mL biotin-labeled probes to hybridize with the membrane cross-linked RNAs. A Biotin Chromogenic Detection Kit (Thermo Fisher Scientific^TM^) was used to detect biotin-labeled probes.

### 2.10 Quantitative real-time PCR (qRT-PCR) analysis

Total RNA was treated with RNase-free DNase I (TaKaRa) at 37°C for 30 min to remove contaminating genomic DNA. DNase was inactivated and removed by phenol extraction. cDNA was synthesized using 1 μg of DNase-treated RNA, 20 pmol of 3′ primer (Supplementary Table S1), and the RT-Kit (BioFact^TM^). qRT-PCR was performed in a 20 µL volume with 2× Real-Time PCR Master Mix (BioFact^TM^). Five microliters of 10-fold diluted cDNA and 10 pmol of primer pairs (Supplementary Table S1) were mixed in a 20 µL reaction. qRT-PCR was performed on a QuantStudio 3 real-time PCR instrument (Applied Biosystems) as described previously (Choi et al., 2020a). The *gapA* gene served as an endogenous control for calculating the relative quantification (RQ) values of *nhaA*, *osmC*, *lpxC*, *waaQ*, *wzx*, *flhD*, *fliA*, and *fliC*. Data were normalized to 23S or 16S rRNA to calculate the RQ values of the pre-23S or pre-16S rRNA. The primers used for qRT-PCR analysis are listed in Supplementary Table S1.

### 2.11 β-Galactosidase assay

The pRS414 derivatives were introduced into ESC69 transformants harboring pACYC184, pACYC184BipA, or pBIS07-1. The cells were incubated at 37°C or 20°C in the early exponential phase and harvested by centrifugation. The pellets were resuspended in 400 µL of Buffer Z (60 mM Na_2_HPO_4_.2H_2_O, 40 mM NaH_2_PO_4_·H_2_O, 10 mM KCl, 1 mM MgSO_4_.7H_2_O, and 50 mM BME), followed by the addition of 4 µL of chloroform and 40 µL of 0.1% SDS. After incubation at 37°C for 5 min, 80 µL of *o*-nitrophenyl-β-D-galactoside (4 mg/mL in H_2_O) was added to each sample. The mixture was incubated for 1–3 min at 25°C, and then 160 µL of 1 M Na_2_CO_3_ was added to terminate the reaction. After centrifugation, the absorbance of the supernatant was measured at 420 and 550 nm using a Multiskan^TM^ GO (Thermo Fisher Scientific^TM^) Microplate Spectrophotometer. β-Galactosidase activity is expressed in Miller units (Miller, 1972).

### 2.12 Protein overexpression and purification

BL21 (DE3) cells harboring pET28NhaR or pET28NhaR_Q35AQ40A_ were incubated in 1 L of LB medium containing Kan at 37°C until OD_600_ of 0.6, and isopropyl β-D-thiogalactopyranoside (IPTG) was added to a final concentration of 0.4 mM. After further incubation at 37°C for 2 h, the cells were harvested by centrifugation and resuspended in 40 mL of Buffer A [20 mM Tris-HCl (pH 8.0), 500 mM KCl, and 5 mM BME] containing 10 mM imidazole and 10 μg/mL lysozyme. After incubation on ice for 20 min, cells were disrupted by sonication. The supernatant obtained after centrifugation at 16,000 ×*g* for 30 min at 4°C was applied to a Ni-NTA agarose resin (QIAGEN) pre-equilibrated with 10 column volumes (CVs) of Buffer A containing 10 mM imidazole. The column was then washed with 20 CVs of Buffer A containing 20 mM imidazole, followed by washing with 20 CVs of Buffer A containing 60 mM imidazole. Wild-type or mutant His_6_-NhaR proteins were eluted with Buffer A containing 400 mM imidazole. The eluted fractions containing His_6_-NhaR were combined and dialyzed overnight in Buffer B [20 mM Tris-HCl (pH 8.0), 100 mM KCl, 15 mM BME, 1 mM EDTA, and 10% glycerol] at 4°C overnight. To purify His_6_-Fis proteins, BL21 (DE3) cells harboring pET28Fis were grown in 1 L of LB medium containing Kan at 37°C till OD_600_ of 0.8–0.9, post which, IPTG was added to a final concentration of 1 mM. The cultures were further incubated for 3 h. After harvesting, cell lysis and protein purification were performed as previously described (Yamamoto et al., 2005).

### 2.13 Electrophoretic mobility shift assay (EMSA)

DNA fragments were amplified by PCR using the primers listed in Supplementary Table S1 and purified after electrophoresis. The purified His_6_-NhaR protein was mixed with DNA (10 ng) in a 10 μL reaction containing 20 mM Tris-HCl (pH 8.0), 100 mM NaCl, 1 mM dithiothreitol (DTT), 10% glycerol, and 125 μg/mL BSA. Binding assays of His_6_-Fis were conducted in 10 mM Tris-HCl (pH 8.0), 150 mM NaCl, 1 mM DTT, and 10 mM MgCl_2_. After incubation for 20 min at 25°C, the formation of the protein-DNA complex was analyzed by 4% native polyacrylamide gel electrophoresis in 0.5× TBE buffer. The gels were then stained with DNA SafeStain (Lamda Biotech) and visualized using Azure C200 (Azure Biosystems).

### 2.14 Statistical analysis

The results are presented as mean ± S.D. of three independent experiments. The data were analyzed using an unpaired two-tailed *t-*test. Statistical significance levels were indicated by: NS, non-significant; *p < 0.05; **p < 0.01; ***p < 0.001.

## 3 Results

### 3.1 NhaR, a suppressor recovering cold sensitivity of the *bipA*-deleted *Escherichia coli*

**FIGURE 1:**
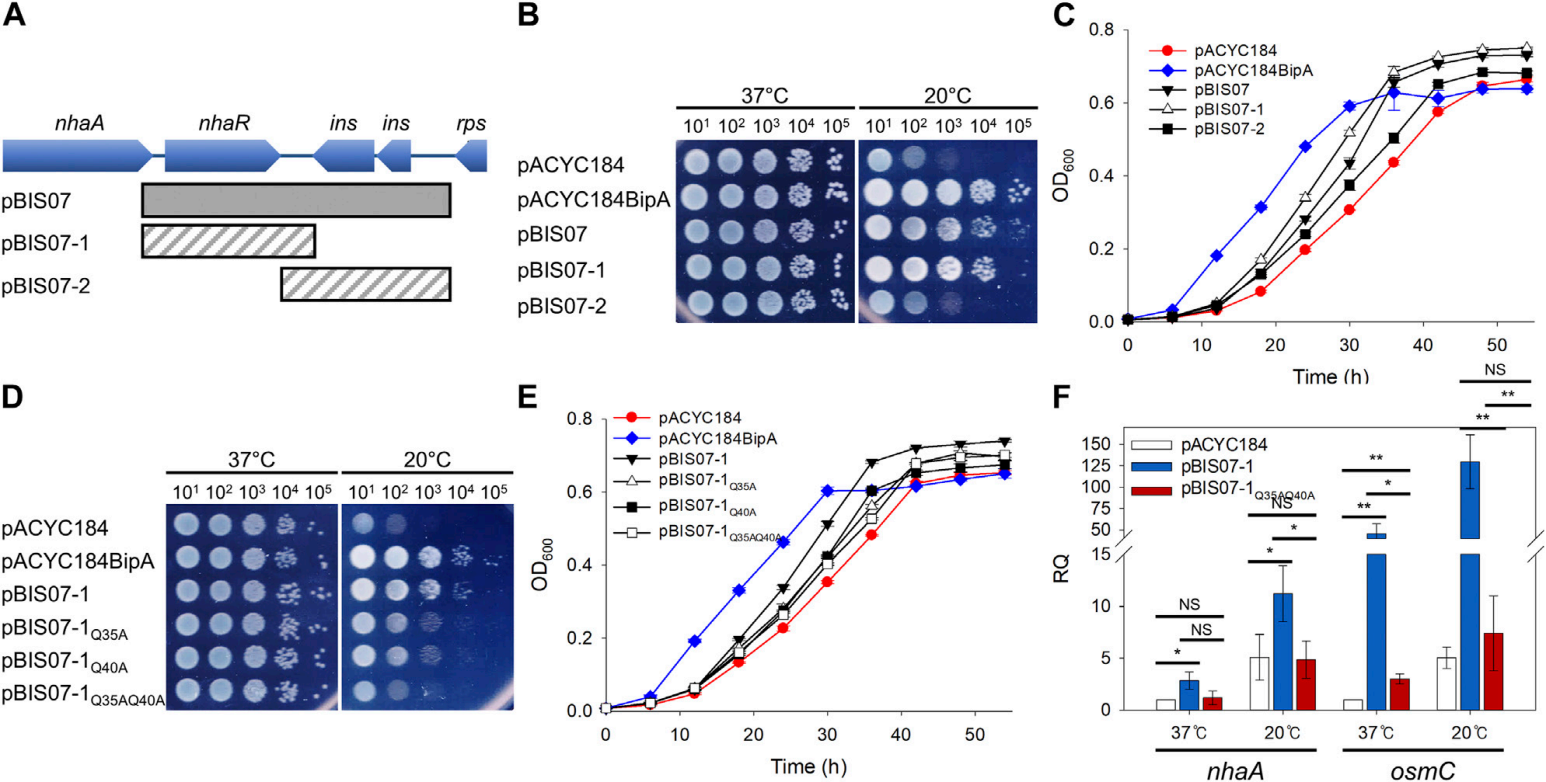
Identification of a suppressor that recovered growth defects of the ESC19 at 20°C. **(A)** Schematic diagram of the genomic locus containing the suppressor gene. The gray square box represents the suppressor clone, pBIS07, isolated from library screening. The diagonal striped boxes represent the truncated clones, pBIS07-1 and pBIS07-2. **(B** and **C)** Recovery of the cold-sensitive phenotype of the *bipA*-deleted strain (ESC19) by pBIS07 or its derivatives on LB agar plates **(B)** or in LB liquid medium **(C)**. The ESC19 cells were transformed with pACYC184, pACYC184BipA, pBIS07, pBIS07-1 and pBIS07-2. The ESC19 transformants were grown in LB medium supplemented with Kan and Cm. Overnight cultures were serially diluted and spotted on LB agar plates supplemented with the same antibiotics, followed by incubation at 37°C or 20°C. To measure growth curves, the overnight cultures of transformants were diluted 10^2^-fold into fresh LB medium and grown at 20°C for 54 h. At every 6 h, the OD_600_ of 5-fold diluted cultures was measured. **(D** and **E)** The suppressive effects of mutants NhaR on growth defect of the strain ESC19 on LB solid medium **(D)** or in LB liquid medium **(E)** at 20°C. **(F)** The transcriptional activity of wild-type and mutant NhaR. The ESC61 cells transformed with pACYC184, pBIS07-1, and pBIS07-1_Q35AQ40A_ were incubated at 37°C or 20°C to the early exponential phase. RNAs were extracted and subjected to qRT-PCR. Error bars represent S.D.

In our previous studies, to understand the function of BipA and to identify its functionally associated partner(s), we performed genomic library screening and isolated clones recovering the growth defect of the *bipA*-deleted *E. coli* strain (ESC19) at low temperature (Choi et al., 2020a; Choi et al., 2020b). Among them, overexpression of *rplT* encoding the r-protein L20 or *yebC* encoding a putative transcriptional regulator partially suppressed defects in ribosome assembly or surface polysaccharide biosynthesis of ESC19, respectively. In the present study, we investigated another positive suppressor clone, pBIS07 containing the *nhaR*-*insB1*-*insA* genomic DNA fragment. NhaR is a Na^+^-responsive transcriptional regulator belonging to the LTTR family, which stimulates the transcription of *nhaA*, *osmC*, and *pgaABCD* genes (Rahav-Manor et al., 1992; Toesca et al., 2001; Goller et al., 2006). *insB1* and *insA* encode the transposase of the insert sequence IS1 (Sekino et al., 1995). To identify *bona fide* suppressor gene(s) in pBIS07, truncated clones pBIS07-1 and pBIS07-2 were constructed using pBIS07 as a template (Figure 1A). ESC19 cells harboring pACYC184, pACYC184BipA, pBIS07, pBIS07-1, and pBIS07-2 were spotted on LB plates containing Kan and Cm and incubated at 37°C or 20°C. As shown in Figure 1B, ESC19 cells harboring pACYC184 exhibited growth defects at low temperature, which were complemented or suppressed by transformation with pACYC184BipA or pBIS07, respectively. ESC19 cells transformed with pBIS07-1 containing only *nhaR* recovered growth to the same extent as pBIS07, whereas pBIS07-2 did not retain the suppression activity. This suppression was further confirmed by monitoring the growth curves of the transformants in liquid LB medium (Figure 1C). Of note, the lag phase of ESC19 cells harboring pACYC184 was much longer than that of the complemented strain or cells harboring pBIS07 and pBIS07-1. Moreover, in the exponential phase, the growth rate of the ESC19 cells overexpressing *nhaR* appeared to be even higher than that of the complemented cells carrying pACYC184BipA. These results indicate that *nhaR* on pBIS07 is indeed a suppressor gene that relieves the cold-sensitive phenotype of ESC19.

As mentioned above, NhaR consists of two domains, an N-terminal DNA-binding domain with an HTH motif and a C-terminal co-inducer (Na^+^)-binding domain (Rahav-Manor et al., 1992). To investigate whether the suppressive effect of NhaR relies on its function as a transcriptional regulator, we chose the highly conserved Gln residues (Q) at positions 35 and 40, subsequently mutated with Ala (A) in the HTH motif to disrupt DNA-binding activity (Supplementary Figure S1A). The suppression activities of NhaR mutants were examined by spotting assay and growth curve monitoring as described above. As shown in Figure 1D, the suppressive activity of NhaR was marginally reduced by a single mutation, which was further deteriorated by the double mutant NhaR_Q35AQ40A_. This result was also confirmed in the liquid medium, thus confirming suppression at the protein level (Figure 1E). This reduced or lost suppression by mutant NhaR was not due to its growth-inhibitory effect, as the overexpression of mutant *nhaR* in wild-type cells did not affect growth (Supplementary Figure S1B). Next, to further ascertain the loss of DNA-binding activity of NhaR_Q35AQ40A_, we carried out qRT-PCR analysis to quantify the mRNA levels of *nhaA* and *osmC*. ESC61 (*nhaR*::kan) cells transformed with pACYC184, pBIS07-1, or pBIS07-1_Q35AQ40A_ were incubated in the early exponential phase, and total RNAs was extracted, followed by qRT-PCR. At both temperatures, deletion of *nhaR* led to a reduction in the levels of the two mRNAs, compared to those of the complemented cells, and the cells harboring pBIS07-1_Q35AQ40A_ failed to recover the transcriptional levels of these genes (Figure 1F). Taken together, our findings demonstrate that NhaR, as a transcriptional regulator, is responsible for this suppression.

### 3.2 Effect of NhaR on surface polysaccharides synthesis in the strain ESC19

**FIGURE 2:**
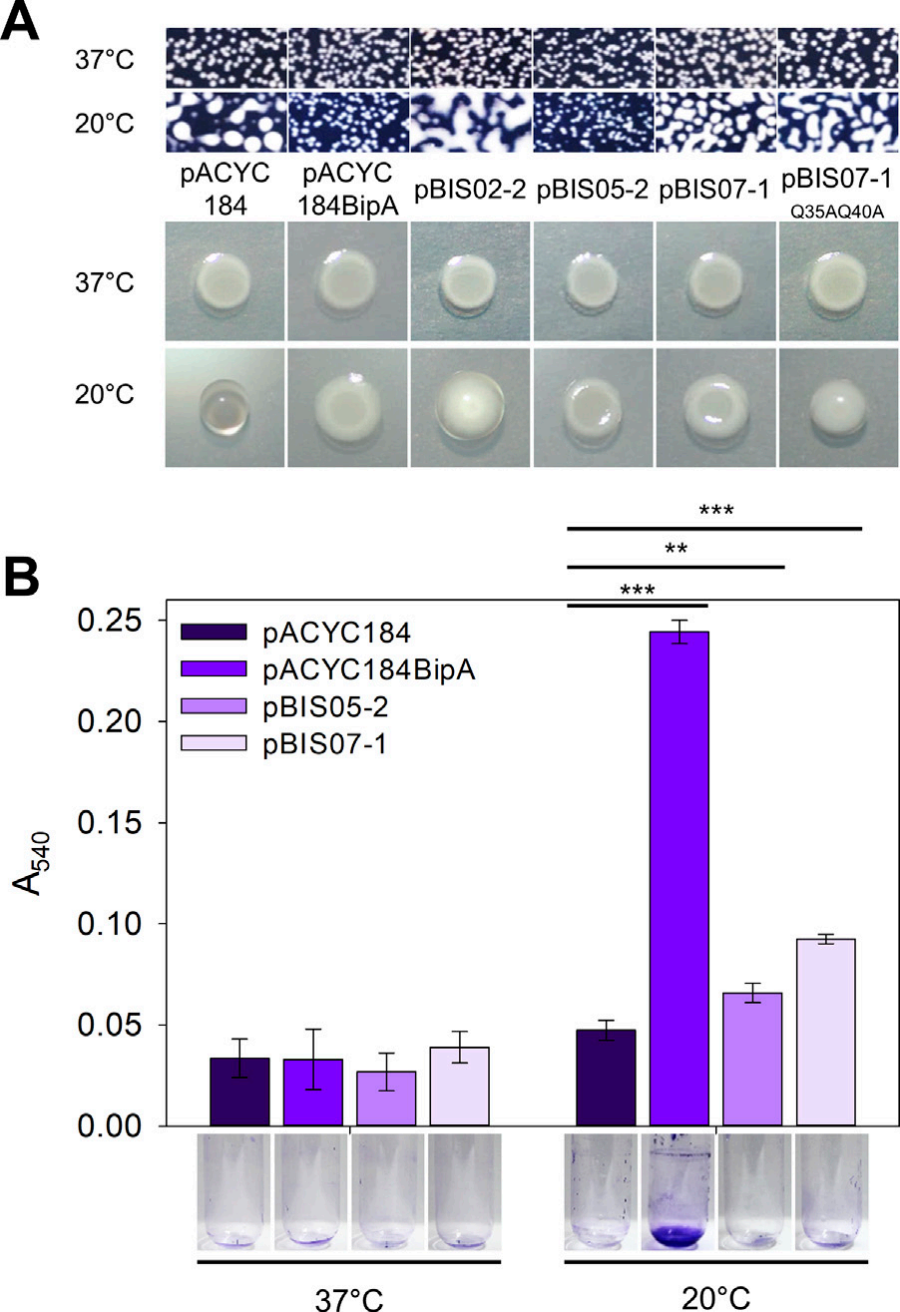
Suppressive effect of NhaR on capsule and biofilm. **(A)** Colony morphologies of the ESC19 transformants. Overnight cultures of ESC19 cells transformed with pACYC184, pACYC184BipA, pBIS02-2, pBIS05-2, pBIS07-1, or pBIS07-1_Q35AQ40A_ were diluted with LB medium and spread on LB agar plates containing appropriate antibiotics, followed by incubation at 37°C or 20°C (upper panel). Macrocolony assays of the same ESC19 transformants as shown in the upper panel. 3 μL of the diluted cultures was spotted on LB agar plates supplemented with appropriate antibiotics and incubated at 37°C or 20°C (lower panel). **(B)** The ESC19 transformants were grown in 2.5 mL of LB medium for 24 h at 37°C and 48 h at 20°C. The culture was discarded, and the biofilm remaining on the surface of the glass tube was stained with 0.1% crystal violet. The absorbance of the dissolved crystal violet was measured at 540 nm. Error bars represent S.D.

As mentioned above, two suppressors, L20 and YebC, have discrete suppression mechanisms that recover ribosome biogenesis and surface polysaccharides, respectively (Choi et al., 2020a; Choi et al., 2020b). Therefore, to understand the possible suppression mechanism of NhaR, we investigated phenotypic alterations in surface polysaccharides in ESC19 cells harboring pBIS07-1. First, we examined capsule production in ESC19 transformants with pACYC184, pACYC184BipA, pBIS02-2, pBIS05-2, pBIS07-1, and pBIS07-1_Q35AQ45A_. pBIS02-2 (encoding *rplT*) and pBIS05-2 (encoding *yebC*) were used as negative and positive controls, respectively, for capsule observation. The transformants were spread on LB agar plates containing Kan and Cm, followed by incubation at 37°C or 20°C. At the optimal growth temperature, small non-mucoid colonies were observed in all the transformants (upper panel in Figure 2A). However, at 20°C, ESC19 cells with pACYC184 formed mucoid and tended to merge between colonies, presumably due to the overproduction of capsular polysaccharides, whereas the complemented cells formed smaller non-mucoid colonies. The morphology of ESC19 cells with pBIS07-1 appeared to be intermediate between that of pACYC184 and pACYC184BipA with mucoid and larger forms, implying that NhaR minimally downregulates the expression of capsule-related genes.

These morphological changes were further visualized by presenting the three-dimensional structures of the macrocolonies (lower panel in Figure 2A). The same transformants were spotted onto LB agar plates and incubated at 37°C or 20°C. All transformants formed flat macrocolonies at 37°C. The ESC19 cells harboring pACYC184 were transparent and had a convex shape at 20°C due to the accumulated capsule. In contrast, transformants with pACYC184BipA had a flat and opaque morphology at 20°C, similar to those at 37°C. The morphologies of cells expressing L20 and YebC were similar to those of transformants with pACYC184 and pACYC184BipA, respectively. In the case of NhaR-suppressed cells, macrocolony morphology was opaque and less protuberant compared to that of ESC19 cells with pACYC184, albeit not as much as that of the YebC-suppressed cells, implying a partial suppression effect on capsule overproduction. As expected, cells expressing NhaR_Q35AQ40A_ were defective in capsule suppression and formed a convex colony. Notably, as the strain ESC61 did not produce excessive capsules, and the mucoid morphology of the strain ESC62 (Δ*bipA*, *nhaR*::kan) was almost similar to that of ESC19 (Supplementary Figure S2), we speculate that NhaR plays a minor role in the repression of capsule production at 20°C compared to BipA.

As mentioned above, the deletion of *bipA* hampers biofilm formation in *E. coli*, as well as in bacteria such as *Bordetella holmesii*, *Pseudomonas aeruginosa*, and *Vibrio cholerae* (Neidig et al., 2013; Hiramatsu et al., 2016; Del Peso Santos et al., 2021). The overproduced colanic acid capsule shields bacterial surface adhesins and antagonizes the attachment stage during biofilm development (Schembri et al., 2004).

Therefore, we investigated whether NhaR partially suppresses capsule overproduction, leading to changes in biofilm formation. ESC19 transformants harboring pACYC184, pACYC184BipA, pBIS05-2, and pBIS07-1 were grown in LB medium at 37°C or 20°C with shaking. The biofilm formed on the inner surface of the glass tubes was quantified using crystal violet staining. As shown in Figure 2B, the amount of biofilm formed by the ESC19 cells with pACYC184 decreased 0.19-fold compared to that of the ESC19 cells with pACYC184BipA at 20°C. Notably, the biofilm formation of the ESC19 transformant with pBIS07-1 was slightly higher than that of cells harboring pBIS05-2. These results suggest that overexpression of *nhaR* partially recovered the biofilm formation ability of strain ESC19 at 20°C.

### 3.3 Increased bile salt resistance and motility by the deletion of *nhaR*

**FIGURE 3:**
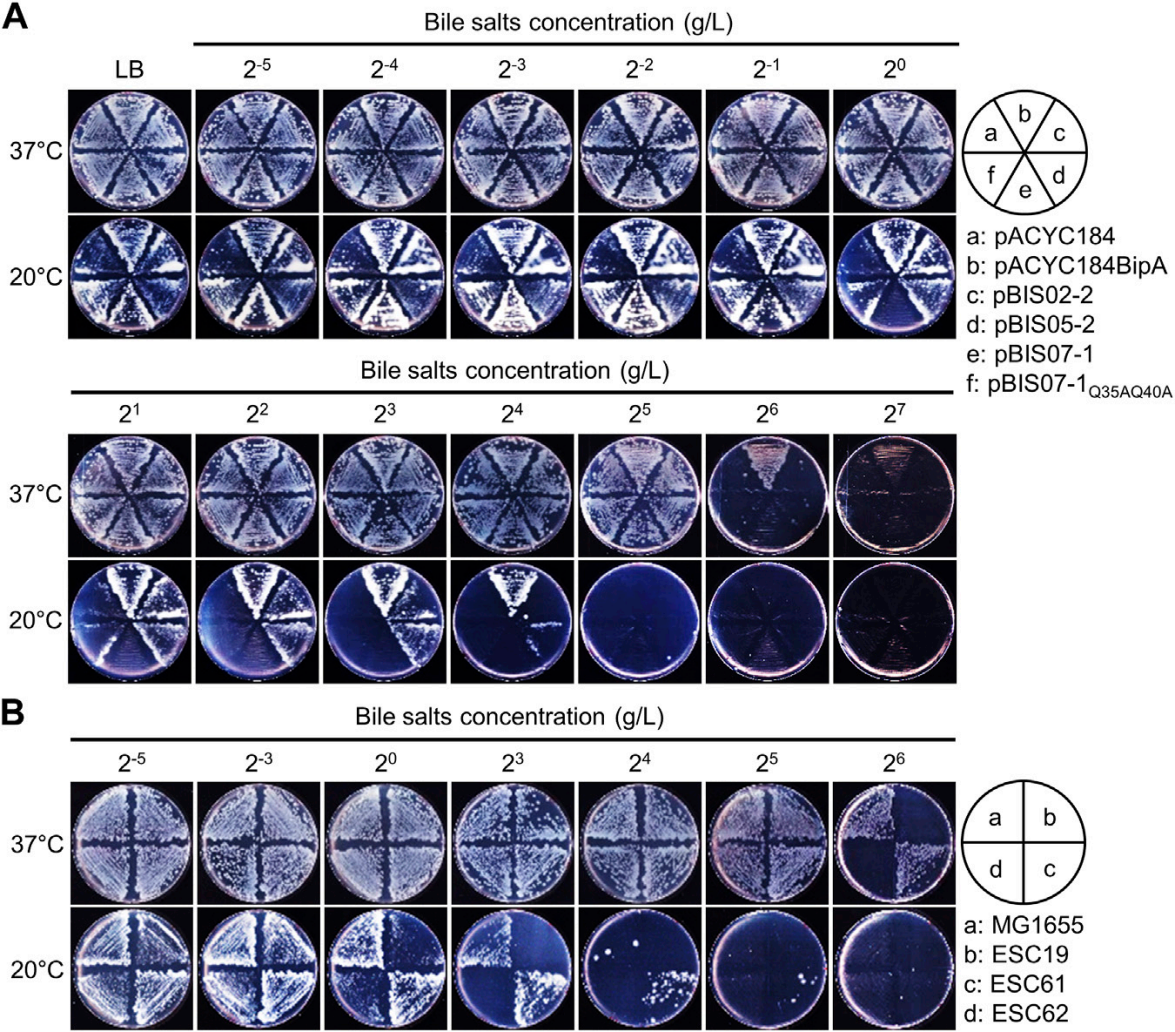
Bile salts sensitivity of the suppressed cells. **(A and B)** Bile salts sensitivity of the ESC19 transformants **(A)** and MG1655, ESC19, ESC61, and ESC62 strains **(B)**. Overnight cultures of ESC19 transformants were diluted to OD_600_ = 0.2 with LB medium, followed by 10^2^-fold dilution. 2.5 µL of the diluted cultures were streaked onto LB plates supplemented with appropriate antibiotics and various concentrations of bile salts, as presented above. The plates were incubated at 37°C or 20°C.

In ESC19 at 20°C, the impaired LPS synthesis accumulating the LPS core precursor transmits the signals of the Rcs pathway, leading to the overproduction of capsular polysaccharides (Weilbacher et al., 2003; Wall et al., 2018). Based on the results shown in Figure 2, it was expected that suppressive NhaR could repair the defective LPS synthesis of ESC19. Therefore, we performed a bile salt sensitivity assay using NhaR-suppressed cells. The ESC19 transformants were streaked on LB agar plates supplemented with 2^−5^ to 2^7^ g/L of bile salts, to which cells with heptose-less LPS became sensitive (Picken and Beacham, 1977). As shown in Figure 3A, at both temperatures, ESC19 cells harboring pACYC184 were more sensitive to bile salts (2^6^ g/L at 37°C and 2^2^ g/L at 20°C) than were ESC19 cells harboring pACYC184BipA (2^7^ g/L at 37°C and 2^5^ g/L at 20°C), indicating a bile salt-sensitive phenotype of ESC19 even at 37°C. Notably, at 20°C, cells harboring pBIS02-2 or pBIS05-2 were more resistant to bile salts, forming colonies similar to the complemented cells at 2^3^ and 2^4^ g/L, respectively. These results suggest that YebC can alleviate LPS impairment better than L20. However, the transformant with pBIS07-1 showed almost the same sensitivity as did the transformant with pACYC184 (2^0^–2^2^ g/L at 20°C). At 2^0^ g/L, overexpression of wild-type *nhaR* inhibited colony formation, whereas cells expressing NhaR_Q35AQ40A_ could still form colonies, implying that the suppressive NhaR may function as a negative regulator of LPS synthesis and that LPS is critical for suppression. To further investigate the role of NhaR in LPS, the same experiment was performed using the *nhaR*-deleted strain (Figure 3B). Strain ESC61 became more resistant to bile salts (2^5^ g/L) than MG1655 cells (2^4^ g/L) at 20°C. This phenotypic difference was not based on increased transcripts of the LPS-synthesizing genes in the ESC61 cells (Supplementary Figure S3), thus, further investigation is necessary to elucidate a possible role of NhaR on LPS regulation.

**FIGURE 4:**
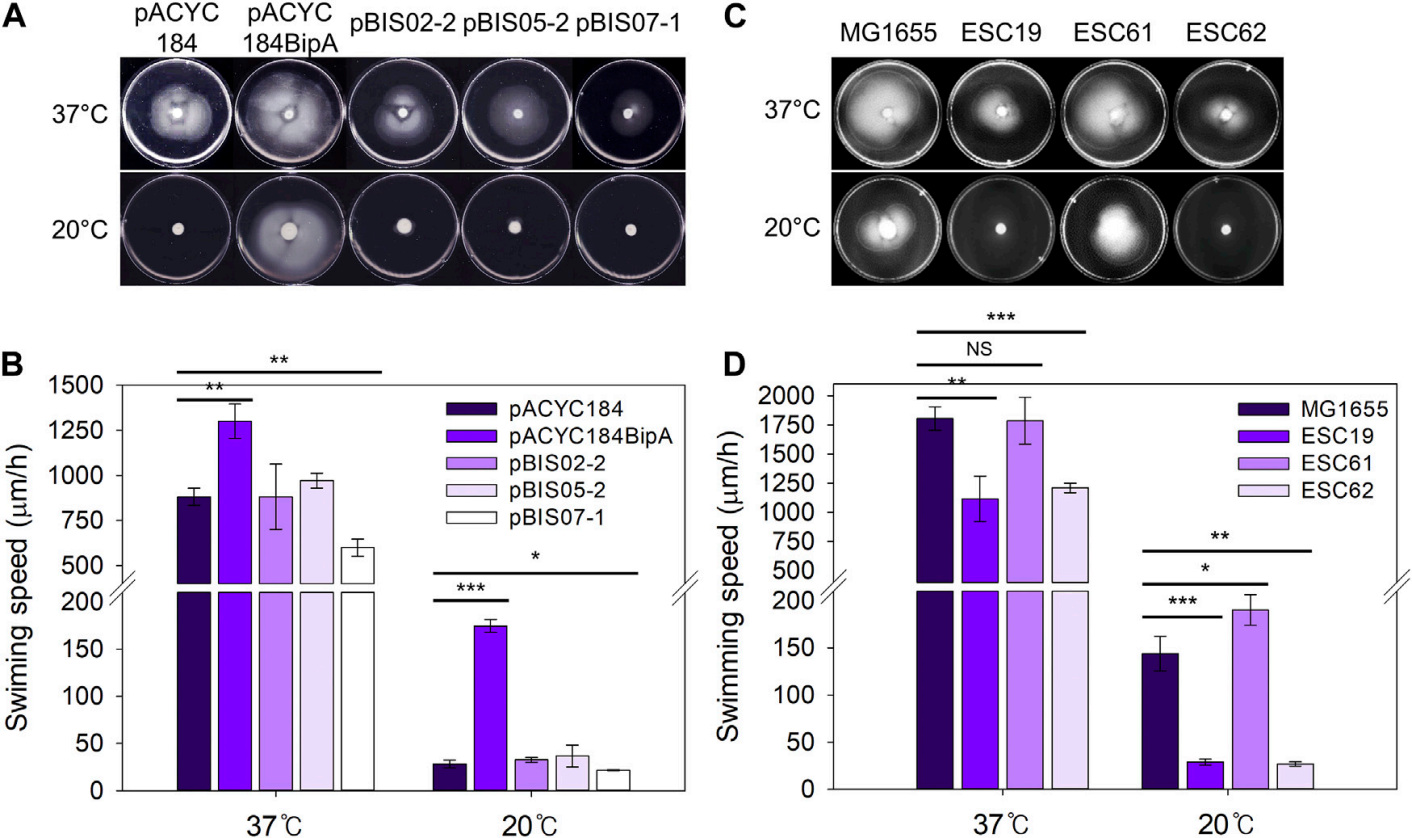
Effect of NhaR on swimming motility. **(A)** The ESC19 cells transformed with pACYC184, pACYC184BipA, pBIS05-2, and pBIS07-1 and **(C)** MG1655, ESC19, ESC61, and ESC62 cells were incubated until OD_600_ reaches 0.5 at 37°C. The cells were harvested by centrifugation at 900 *g* followed by resuspension in LB medium. 5 μL of suspension was injected into LB plates containing Kan, Cm, and 0.3% bacto agar, and the plates were grown at 37°C or 20°C. These experiments were performed three times independently. **(B** and **D)** Comparison of swimming speed plotted as a bar diagram. To calculate swimming speed (μm/h), the average migration distance (μm) in **(A)** and **(C)** was divided by the incubation time (h). Error bars represent S.D.

As mentioned above, the deletion of *bipA* in *E. coli* reduced swimming motility at both 37°C and 20°C (Choi and Hwang, 2018), and recently, the deletion of *napA*, encoding a homolog of NhaA, was reported to reduce motility in *Helicobacter pylori* (Masaaki et al., 2021). Therefore, to examine the involvement of NhaR in swimming motility, the same transformants and deletion strains as those in Figure 3 were injected into LB plates containing 0.3% agar and incubated at 37°C or 20°C. At both temperatures, the swimming speed of the ESC19 cells with pACYC184 was significantly slower (0.67- and 0.16-fold at 37°C and 20°C, respectively) than that of the complemented cells, and neither pBIS02-2 nor pBIS05-2 recovered the impaired motility of ESC19 cells (Figures 4A,B) (Choi et al., 2020a). Notably, at both temperatures, the motility of the cells harboring pBIS07-1 was reduced (0.68-fold at 37°C and 0.77-fold at 20°C) compared to that of the cells harboring pACYC184, implying that overexpression of *nhaR* may play a negative role in cellular motility at either temperature. To examine the direct role of NhaR in motility, the swimming motilities of the strains MG1655, ESC19, ESC61, and ESC62 were analyzed under the similar conditions. At 37°C, the swimming speeds of MG1655 and ESC61 cells were not significantly different; however, the motility of the ESC61 cells was moderately faster (1.32-fold) than that of the wild-type at 20°C, suggesting that NhaR might be involved in the downregulation of flagella-mediated motility (Figures 4C,D).

This possibility was further verified by analyzing transcript levels of flagella-related genes (*flhD, fliA*, and *fliC*) in wild-type and ESC61 cells. As shown in Supplementary Figure S3, the ESC61 strain increased the mRNA levels of *flhD* (1.42-fold), *fliA* (2.01-fold), and *fliC* (2.05-fold) at 20°C, confirming its negative regulation on motility.

### 3.4 Recovery of ribosome assembly defects in the strain ESC19 by NhaR

A comparison of the growth recovery by the three suppressors L20, YebC, and NhaR revealed that similar to L20, NhaR resulted in a faster growth restoration than did YebC, implying a ribosome recovery in the NhaR-suppressed cells (Supplementary Figure S4). Therefore, we examined whether the overexpression of *nhaR* suppresses ribosomal defects in ESC19 at low temperature. Sucrose density gradient sedimentation was performed using ESC19 cells transformed with pACYC184, pACYC184BipA, pBIS07-1, and pBIS07-1_Q35AQ40A_. The polysome and subunit profiles of ESC19 cells harboring pBIS02-2 or pBIS05-2 were simultaneously analyzed as positive and negative controls, respectively.

**FIGURE 5:**
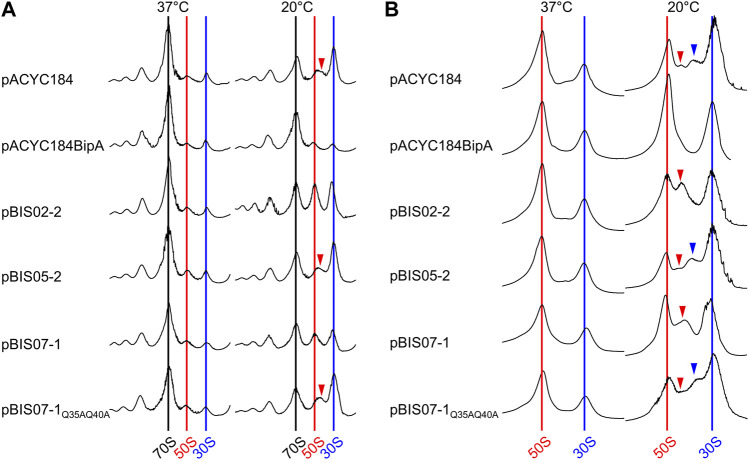
Restoration of ribosome assembly defects by overexpression of *nhaR*. Polysome **(A)** and subunit **(B)** profiles of ESC19 cells harboring pACYC184, pACYC184BipA, pBIS02-2, pBIS05-2, pBIS07-1, and pBIS07-1_Q35AQ40A_. The cell lysates were prepared from ESC19 transformants grown at 37°C or 20°C to the early exponential phase and subjected to sucrose gradient density sedimentation. Arrow heads indicate immature 50S ribosomal particles.

As observed in our previous studies (Choi and Hwang, 2018), at the optimal growth temperature (37°C), all transformants displayed a normal distribution of ribosomal particles with high 70S ribosomal peaks and lower ribosomal subunit peaks (left panel in Figure 5A). To the contrary, at 20°C, ESC19 cells harboring pACYC184 showed a significant reduction in 70S ribosomes and accumulation of free ribosomal subunits (right panel in Figure 5A). In addition, an abnormal peak was observed between the two ribosomal subunits (red arrowheads in Figure 5A), which is consistent with previous studies (Choi and Hwang, 2018; Gibbs and Fredrick, 2018; Goh et al., 2021). This profile was also observed in the cells transformed with pBIS05-2. These abnormal profiles caused by *bipA* deletion at 20°C were complemented by the introduction of pACYC184BipA, and the aberrant 50S ribosomal particles disappeared in cells harboring pBIS02-2 and pBIS07-1, whose partial suppression led to slightly higher ribosomal subunit peaks than those in the complemented cells*.*

Subsequently, these abnormalities in the 50S ribosomal subunit were analyzed at a lower sucrose gradient concentration. In the transformants grown at 37°C, both the 50S and 30S ribosomal subunits were well separated without a noticeable aberrant peak. However, ESC19 cells harboring pACYC184 and pBIS05-2 grown at 20°C showed two additional peaks at aberrant positions between the 50S and 30S ribosomal subunits (red and blue arrowheads in Figure 5B) with a concomitant reduction in the 50S ribosomal subunit. These abnormal peaks appeared only at the lower sucrose gradient concentration, and lighter particles (blue arrow) had been designated as 44S particles which is missing the r-protein L6 (Choi and Hwang, 2018). In the subunit profiles of ESC19 cells with pBIS02-2 and pBIS07-1, only a single precursor particle appeared adjacent to the 50S ribosomal subunit peak, with increased normal 50S ribosomal subunits (red arrowhead in Figure 5B). (Goh et al., 2021) Based on these findings, we confirmed that YebC could not resolve ribosomal defects in the strain ESC19. Notably, ESC19 cells expressing either L20 or NhaR exhibited almost identical polysome and subunit profiles, implying that suppressive NhaR may be able to promote ribosome biogenesis at low temperature. Further, the expression of NhaR_Q35AQ40A_ failed to recover ribosomal assembly defects in ESC19 cells.

### 3.5 Reduction of rRNA precursors in the suppressed cells

During rRNA maturation, a series of endo- and exo-ribonucleases, and modification enzymes are required to produce mature and functional rRNA. Ribosome assembly defects are generally accompanied by rRNA processing abnormalities (Shajani et al., 2011). 7 nt at 5′and 7–9 nt at 3′ends of pre-23S rRNA sequence and 115 nt at 5′and 33 nt at 3′ends of pre-16S rRNA are removed to produce mature forms (Young and Steitz, 1978; King et al., 1986) (Supplementary Figure S5). It was also reported that the deletion of *bipA* leads to the accumulation of rRNA precursors (Choi et al., 2020b), and upregulation of ribosome maturation factors such as DeaD and RNase R (Goh et al., 2021). Therefore, to investigate whether NhaR restores rRNA processing in ESC19, rRNA from ESC19 cells harboring pACYC184, pACYC184BipA, pBIS02-2, or pBIS07-1 were analyzed by Northern blotting. Probes 23S-M and 16S-M were designed to specifically bind to the internal region of each rRNA, and probes P23S-U, P16S-U, and P16S-D were complementary to the sequences of unprocessed 5′and 3′ends of rRNA precursors (Supplementary Figure S5). The levels of rRNA precursors were normalized by dividing the intensity of the precursor band by that of the band in the internal region.

**FIGURE 6:**
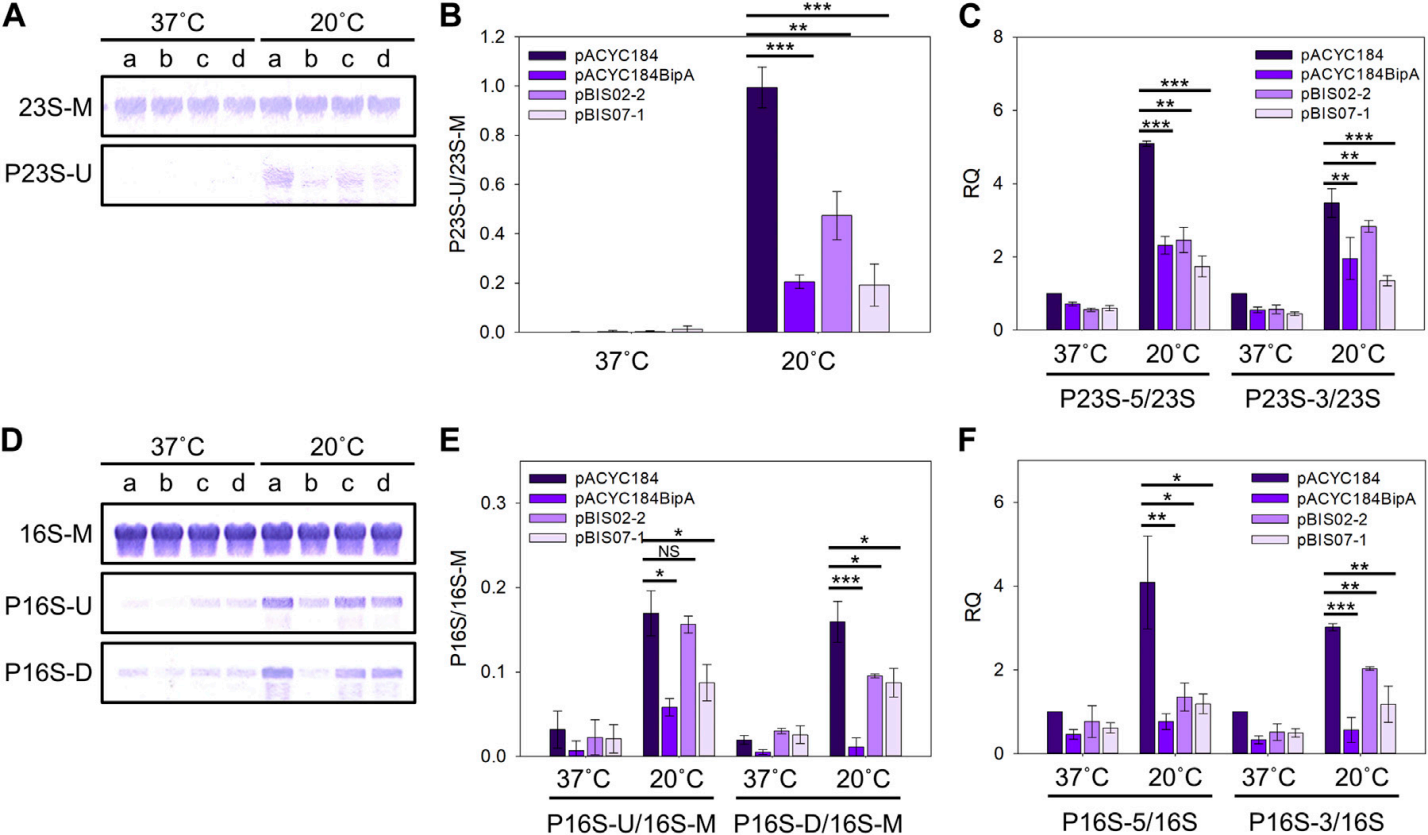
Reduction in the levels of rRNA precursors by overexpression of *nhaR.* **(A** and **D)** Northern blot analyses of 23S **(A)** and 16S **(D)** rRNAs in the ESC19 transformants. Total RNAs extracted from ESC19 cells with pACYC184, pACYC184BipA, pBIS02-2, or pBIS07-1 were subjected to Northern blotting. a, pACYC184; b, pACYC184BipA; c, pBIS02-2; and d, pBIS07-1. **(B** and **E)** Densitometric analyses of 23S **(B)** and 16S **(E)** rRNAs. The blots in **(A)** and **(D)** were quantified using the software ImageJ. **(C** and **F)** qRT-PCR analyses of 23S **(C)** and 16S **(F)** rRNAs in the ESC19 transformants. Total RNAs were prepared from the ESC19 transformed and subjected to qRT-PCR. The 23S and 16S rRNAs served as references for normalization in calculating the relative quantification (RQ) values of unprocessed 23S and 16S rRNAs, respectively. Error bars represent S.D.

At 20°C, the ESC19 cells with pACYC184 accumulated 23S rRNA precursors at a high level (P23S-U, 0.99), which was significantly reduced in the ESC19 cells with pACYC184BipA, pBIS02-2, or pBIS07-1 (pACYC184 BipA, 0.21; pBIS02-2, 0.47; pBIS07-1, 0.19) (Figures 6A,B). In contrast, at 37°C, almost no precursors were detected in any of the transformants. The suppression of 23S rRNA processing by NhaR was further verified by qRT-PCR using primer pairs amplifying the internal region (23S) or unprocessed regions (P23S-5 and P23S-3) (Supplementary Table S1 and Supplementary Figure S5). As shown in Figure 6C, the ratios of P23S-5/23S and P23S-3/23S in ESC19 cells with pACYC184 were increased 5.09- and 3.46-fold, respectively, at 20°C compared to 37°C. At 20°C, the ratios in ESC19 complemented by pACYC184BipA were significantly decreased (P23S-5, 0.46-fold; P23S-3, 0.57-fold) compared to ESC19 with pACYC184. These reductions were also observed in ESC19 cells overexpressing NhaR (P23S-5, 0.34-fold; P23S-3, 0.39-fold), which were lower than the levels of 23S rRNA precursors in ESC19 cells harboring pBIS02-2.

Similar to 23S rRNA, 16S rRNA was typically processed in all transformants at 37°C, leaving a trace amount of precursors. On the contrary, at low temperature, unprocessed 16S rRNA precursors were highly accumulated in ESC19 with pACYC184 (P16S-U, 0.17; P16S-D, 0.16), which was reduced by overexpression of *bipA* (P16S-U, 0.06; P16S-D, 0.01) and *nhaR* (P16S-U, 0.09; P16S-D, 0.09) (Figures 6D,E). The levels of 16S rRNA species were further examined using qRT-PCR. Overexpression of *bipA* and *nhaR* promoted the processing of the 5′and 3′ends of 16S rRNA precursors accumulated in ESC19 at low temperature (Figure 6F). The levels of P16S-5 and P16S-3 decreased 0.29- and 0.39-fold in ESC19 cells transformed with pBIS07-1, respectively, compared to those in ESC19 cells harboring pACYC184. Analysis of 23S and 16S rRNAs revealed that the suppressive activity of NhaR on rRNA processing was slightly higher than that of L20. Our results indicate that overexpressed NhaR in ESC19 can help rRNA processing, consequently reducing abnormal ribosomal particles, as shown in Figure 5.

### 3.6 Alterations in the activities of seven *rrn* promoters by NhaR

Ribosome biogenesis is a complex and energy-consuming process governed by the levels of rRNA and r-proteins and their relative ratios. The expression of rRNA is fine-tuned by transcriptional regulators such as Fis, H-NS, and Lrp (Nilsson et al., 1990; Tippner et al., 1994; Pul et al., 2005). As shown in Figure 1F, NhaR_Q35AQ40A_ lost the transcriptional activation of *nhaA* and *osmC*. Therefore, we rationalized that NhaR, as a transcriptional regulator, might promote rRNA transcription and stimulate ribosome biosynthesis.

**FIGURE 7:**
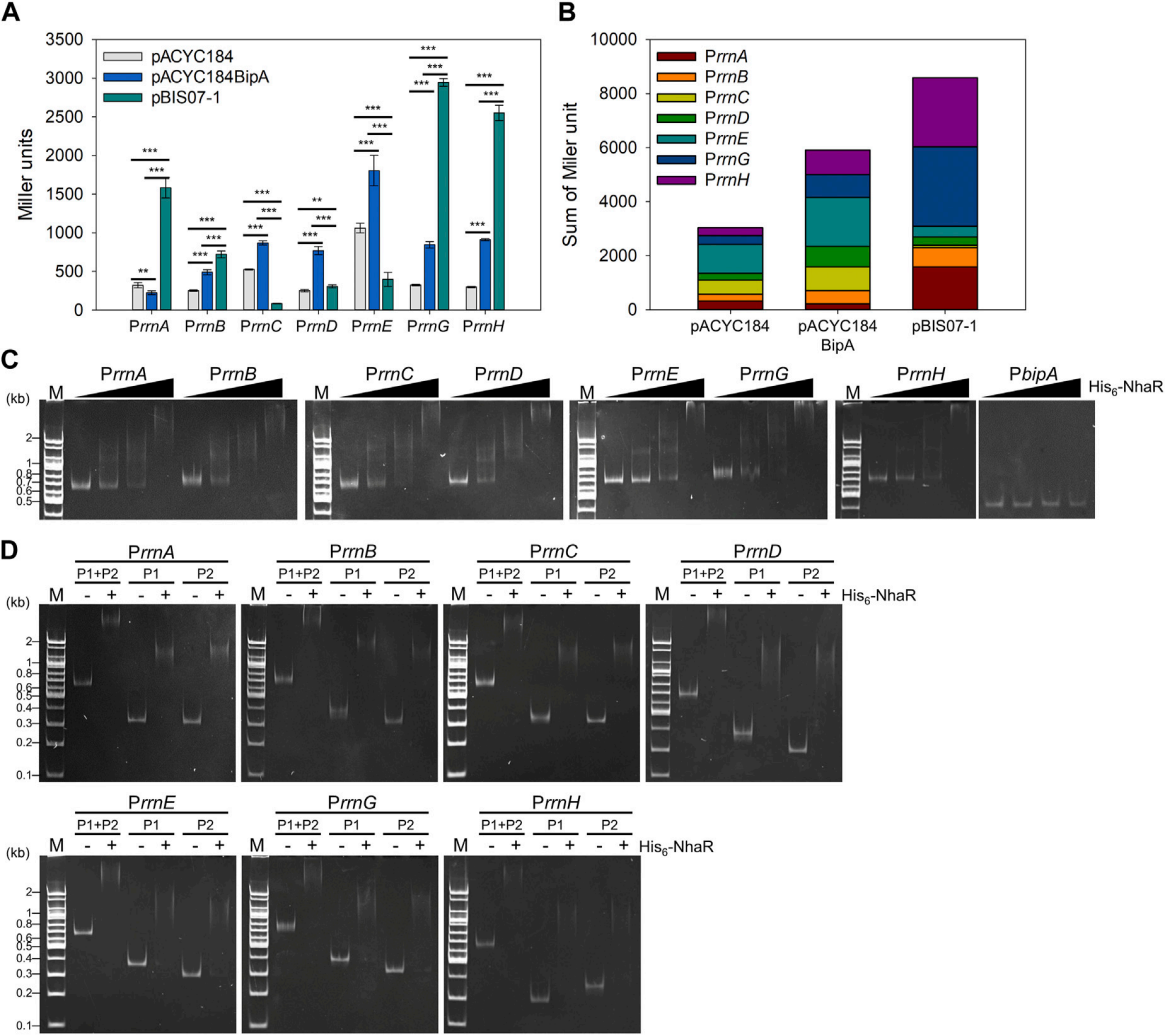
Effect of NhaR on rRNA expression. **(A)** Activities of seven *rrn* promoters in the ESC69 transformants. pRS414 derivatives were introduced into the ESC69 pre-transformed with pACYC184, pACYC184BipA, or pBIS07-1. The transformants were incubated at 20°C to the early exponential phase and then subjected to β-galactosidase assay. Error bars represent S.D. **(B)** Sum of Miler units in each ESC69 transformant shown in **(A)**. **(C)** EMSA analysis using His_6_-NhaR and the promoter DNA fragments of seven *rrn* operons. 10 ng of each promoter DNA fragments of seven *rrn* operons was incubated with increasing concentrations of His_6_-NhaR (0, 150, 300, or 600 nM). The promoter DNA of *bipA* was served as a negative control. **(D)** EMSA analysis of the truncated *rrn* promoter DNA fragments with His_6_-NhaR. 10 ng of DNA fragments were incubated with 300 nM His_6_-NhaR. M, 100-bp DNA ladder (Bioneer).

Thus, we investigated the effect of *nhaR* overexpression on rRNA expression in a *bipA*-deleted strain. To this end, reporter plasmids were constructed by translationally fusing the promoter of seven *rrn* operons (P*rrn*) with *lacZ* (Supplementary Figure S6A) and introduced into ESC69 (Δ*bipA*, *lacZ*::kan) harboring pACYC184, pACYC184BipA, and pBIS07-1. After growing the transformants at 20°C until the early exponential phase, the β-galactosidase activity was measured. As shown in Figure 7A, the expression levels of *rrn* promoters, except for P*rrnA*, were decreased in ESC69 cells containing pACYC184 compared to those in ESC69 cells harboring pACYC184BipA. Notably, when the plasmid pBIS07-1 was introduced, the levels of β-galactosidase expressed from P*rrnA*, P*rrnB*, P*rrnG*, and P*rrnH* increased by 7.07-, 1.47-, 3.49-, and 2.8-fold, respectively, compared to those of ESC69 cells containing pACYC184BipA. Those of P*rrnC*, P*rrnD*, and P*rrnE* were reduced by 0.10-, 0.40-, and 0.22-fold, respectively. Each enzymatic activity from the three transformants harboring pACYC184, pACYC184BipA, or pBIS07-1 was added, and the total activities were compared, as shown in Figure 7B. The sum of activities was higher in the order of pBIS07-1, pACYC184BipA, and pACYC184. These findings suggest that overexpression of *nhaR* can enhance overall rRNA levels, which was also confirmed in the MG1655 background (Supplementary Figure S7). This supports our hypothesis that NhaR, as a transcriptional regulator, might promote rRNA transcription. Furthermore, we observed dramatic transcriptional activations in P*rrnA*, P*rrnG*, and P*rrnH*, and repression of P*rrnC*, P*rrnD*, and P*rrnE*, implying that when cells overexpress NhaR, it might be able to modulate the expression of *rrn* operons to adjust to that specific condition.

Next, we tested whether NhaR directly binds to the promoter of *rrn* operons. In strain MG1655, the respective promoter of *rrn* operons (P*rrn*) has two tandem promoters, upstream P1*rrn* and downstream P2*rrn*, with an interspace of ∼120 bp (Gilbert et al., 1979; Neidhardt et al., 1987) (Supplementary Figure S6A). P1*rrn* is a major promoter responsible for rRNA transcription under exponentially growing optimal conditions (Murray et al., 2003). In contrast, P2*rrn* is less active than P1*rrn* during fast growth but is activated for the basal-level expression of rRNA during stationary or slow growth phases (Murray and Gourse, 2004). First, EMSA assays were performed using purified His_6_-NhaR protein and DNA fragments P*rrn*, P1*rrn* and P2*rrn*, in the presence of 100 mM NaCl. When increasing amounts of His_6_-NhaR (0–600 nM) were added to the reactions, the bands corresponding to the NhaR-P*rrn* complex shifted up at multiple positions in an NhaR-concentration-dependent manner with a concomitant disappearance of free DNA. NhaR did not bind to the promoter of *bipA* (P*bipA*) (Figure 7C). However, this complex formation was disrupted in the presence of His_6_-NhaR_Q35AQ40A_ (Supplementary Figure S6B), indicating direct binding of NhaR to P*rrn*. This specific complex formation was further verified using purified His_6_-Fis protein, which is known to bind upstream of P1*rrn* (Ross et al., 1990), and the promoter DNAs of *nhaA*, *osmC*, and *pgaA* (Carmel et al., 1997; Toesca et al., 2001; Goller et al., 2006; Choi and Hwang, 2018). His_6_-Fis protein was able to bind to P*rrn* DNA fragments (Supplementary Figure S6C), and the P*nhaA*, P*osmC*, and P*pgaA* DNA fragments were shifted up in the presence of His_6_-NhaR (Supplementary Figure S6D). Furthermore, as shown in Figure 7D, His_6_-NhaR was able to bind not only to full-length P*rrn* but also to individual subpromoters P1*rrn* and P2*rrn*. Therefore, our results support the existence of a specific interaction between NhaR and P*rrn*, and it is likely that NhaR may be a novel trans-acting element for the promoter of the seven *rrn* operons in *E. coli*.

### 3.7 Effect of *bipA* or *nhaR* deletion on growth under salt stress condition

**FIGURE 8:**
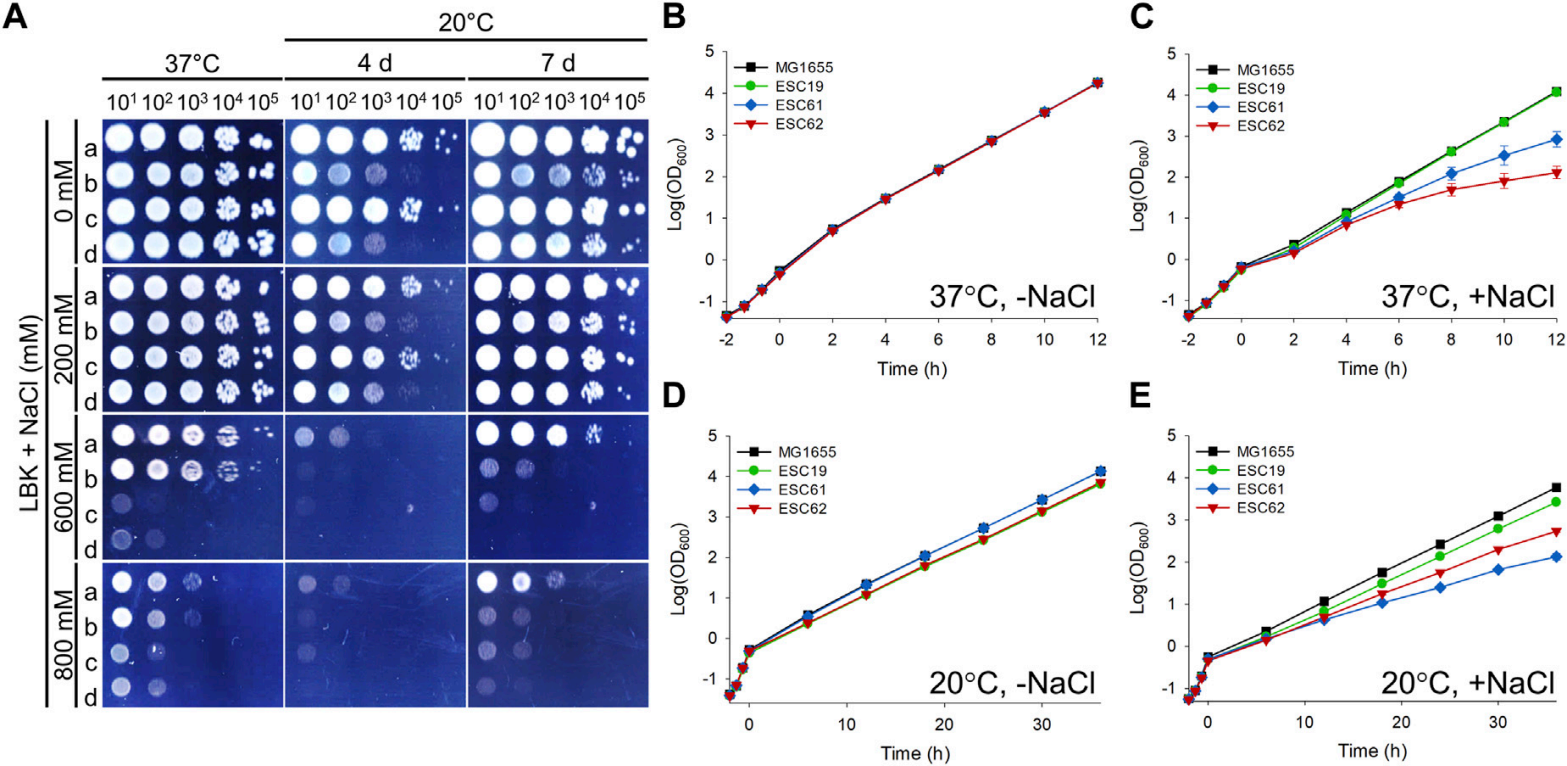
Effects of *bipA* or *nhaR* deletion on growth under high salt conditions. **(A)** Spotting assay at various NaCl concentrations. The overnight cultures of the strains MG1655, ESC19, ESC61, and ESC62 were diluted as described in Figure 1B. 3 μL of each diluted cultures was spotted on LBK agar plates with different NaCl concentrations as presented above, followed by an incubation at 37°C or 20°C. **(B–E)** Growth curves in LBK medium with different concentration of NaCl. The overnight cultures of the strains MG1655, ESC19, ESC61, and ESC62 were inoculated 10^2^-fold in LBK medium, followed by an incubation until the exponential phase at 37°C. The cultures were diluted 5-fold into the LBK medium without NaCl and incubated at 37°C **(B)** or 20°C **(D)**. Exponentially growing cultures were also diluted 5-fold in LBK medium to a final concentration of NaCl of 500 mM **(C)** or 300 mM **(E)** and incubated at 37°C or 20°C, respectively. At every 2 h at 37°C or 6 h at 20°C, the cultures were diluted in the same LBK medium, and the OD_600_ was measured as mentioned above.

So far, the possible roles of NhaR in suppressing *bipA* deletion phenotypes at 20°C have been explored. However, considering the native function of NhaR at high Na^+^ concentrations, the potential role of BipA in salt stress adaptation remains unexplored. Thus, we examined the growth of strains MG1655, ESC19, ESC61, and ESC62 under high salt and basic pH conditions. Overnight cultures were spotted on LBK containing 0, 200, 600, or 800 mM NaCl, followed by incubation at 37°C or 20°C. At 37°C, there was no apparent difference in growth between all strains up to 200 mM NaCl; however, in the presence of 600 or 800 mM NaCl, colony formation of ESC61 and ESC62 was significantly inhibited (Figure 8A). At 20°C, the growth of ESC19 and the *nhaR*-deleted strains (ESC61 and ESC62) appeared to be more inhibited than that of MG1655 cells in a medium containing ≥600 mM NaCl. Next, to clarify the growth of ESC19, ESC61, and ESC62 and to maintain a constant NaCl concentration and pH during cultivation, growth monitoring was carried out in a consecutively diluted fresh medium as described in the “Materials and methods.” As shown in Figure 8B, all the strains showed the same growth in LBK medium at 37°C without NaCl, whereas both ESC61 and ESC62 cells showed growth defects in the presence of 500 mM NaCl. Notably, ESC62 cells showed more severe growth defects than ESC61 cells (Figure 8C). As expected, the *bipA*-deleted strains, ESC19 and ESC62, exhibited slightly lower cell densities due to the cold sensitivity caused by *bipA* deletion at 20°C in LBK medium without NaCl (Figure 8D). As shown in Figures 8D,E, the strains MG1655 and ESC19 showed identical growth in the presence and absence of NaCl; however, in the presence of NaCl at 20°C, the ESC62 cells grew slightly better than the ESC61 cells, whose phenotype was the inverted result of Figure 8C. This is probably due to the protective role of the overproduced colanic acid against high concentrations of NaCl (Chen et al., 2004). These results suggest that NhaR is required for adaptation to high salt concentrations at both temperatures and that BipA may be implicated in adapting to high salt stress even at 37°C.

## 4 Discussion

In this study, we investigated the possible suppression mechanism of NhaR on *bipA* deletion phenotypes and analyzed the impact of NhaR on ribosome assembly and virulence factors, including capsular polysaccharides, biofilms, LPS, and swimming motility. Overexpression of *nhaR* partially restored ribosome biogenesis, capsule production, and biofilm formation, but not LPS synthesis and swimming motility, in ESC19 cells at low temperature.

The various impairments in the ESC19 cells and their suppression by NhaR could be postulated by a global regulatory system, “Rcs (regulator of capsule synthesis) phosphorelay signaling pathway”. In the Rcs two-component system, membrane stress signals caused by osmotic upshift, cold shock, or defects in LPS are sensed by the outer membrane sensor protein RcsF, whose activation triggers the autophosphorylation of the inner membrane protein RcsC (Parker et al., 1992; Sledjeski and Gottesman, 1996; Hagiwara et al., 2003; Majdalani et al., 2005). This phosphate is then transferred to another membrane protein, RcsD, which eventually phosphorylates the cytosolic response regulator, RcsB (Takeda et al., 2001). RcsB forms either a homodimer or heterodimer, depending on its phosphorylation status. Phosphorylated RcsB forms a homodimer or heterodimer with RcsA. The former dimer activates *osmC* expression but represses biofilm formation (Davalos-Garcia et al., 2001), whereas the latter activates colanic acid production (the *cps* genes) (Stout et al., 1991) and represses flagella-mediated motility (the *flhDC* genes) (Francez-Charlot et al., 2003). This RcsB-RcsA heterodimerization is the limiting process, in that, the intracellular level of RcsA is very low due to gene silencing mediated by H-NS at the transcriptional level (Sledjeski and Gottesman, 1996) and proteolysis mediated by Lon protease at the post-translational level, consequently maintaining the low expression of *cps* in wild-type cells (Torres-Cabassa and Gottesman, 1987). Unphosphorylated RcsB forms a heterodimer with various auxiliary proteins, such as BglJ, MatA, and GadE, to regulate the transcription of *bgl*, *gadA/BC*, and *mat*, respectively (Castanie-Cornet et al., 2010; Venkatesh et al., 2010; Pannen et al., 2016).

Assuming that LPS damaged by *bipA* deletion is an initiator of the Rcs pathway, the suppressive NhaR did not restore the LPS core defect, resulting in increased susceptibility to bile salts (Figure 3A). In contrast, the *nhaR*-deleted strain was more resistant to bile salts than the wild-type strain (Figure 3B). We postulate the following three hypotheses: First, the expression of NhaA, the main Na^+^/H^+^ antiporter in *E. coli*, is activated by NhaR (Rahav-Manor et al., 1992), and the structure of NhaA has a unique fold termed as the “NhaA fold” with twelve transmembrane helices (Quick et al., 2021). In a recent report, it was shown that the *Neisseria meningitidis* Na^+^-dependent bile acid symporter (ASBT_NM_) remarkably resembled the NhaA fold (Hu et al., 2011), suggesting that an increased amount of NhaA might cause higher sensitivity to bile salts. UDP-GlcNAc is a common substrate for the synthesis of LPS, PGA, and peptidoglycan (Brozek et al., 1987; Neidhardt et al., 1987; Wang et al., 2004). The expression of *pgaABCD,* responsible for PGA synthesis, is also enhanced by NhaR (Goller et al., 2006). Therefore, it is likely that UDP-GlcNAc is preferentially utilized for PGA synthesis rather than LPS synthesis in suppressed cells, causing sensitivity to bile salts. Finally, we do not exclude the possibility that NhaR directly or indirectly inhibits the expression of AcrAB or EmrAB, which are multidrug efflux pumps discharging bile salts (Thanassi et al., 1997). Notably, we noticed that the overexpression of *rplT* partially suppressed the sensitivity of ESC19 cells to bile salts, albeit not as much as YebC. Bile salts are strong detergents that not only disrupt bacterial membranes, inhibiting bacterial growth or cell lysis (Urdaneta and Casadesus, 2017) but also penetrate the cytoplasm, triggering extensive protein aggregation (Cremers et al., 2014). Of the bile salt-induced aggregated proteins, both r-proteins and translation factors accounted for 47%, indicating that the ribosome and translation machinery are severely damaged in the presence of bile salts (Cremers et al., 2014). Moreover, in *Salmonella enterica* serovar Typhimurium, a high decoding fidelity rpsL* mutant benefits fitness upon bile salt exposure, whereas the error-prone rpsD* mutant has the opposite effect in the presence of bile salts (Lyu and Ling, 2022). This is also applicable to L20-suppressed ESC19 cells at low temperature, even though *bipA* deletion does not influence the fidelity of translation at 37°C (Shoji et al., 2010).

Despite the deterioration of LPS defects by NhaR, the NhaR-suppressed cells seemed to produce slightly less capsule and marginally reduced motility compared to the ESC19 cells harboring pACYC184 at low temperature (Figures 2, Figure 4). This indicates that the overexpression of *nhaR* affects the capsule and motility without respect to Rcs signaling. Thus, at present, we cannot rule out the possibility that NhaR itself may repress the expression of the *cps* gene cluster and flagella-related genes.

For biofilm formation, the minute reduction in colanic acid overproduction by NhaR can contribute to the recovery of biofilm-forming ability because it inhibits bacterial adhesion to solid surfaces at the early stage of biofilm development (Hanna et al., 2003). Moreover, increased biofilm formation is likely a result of transcriptional activation of *pgaABCD* by NhaR. PGA produced from *pgaABCD* serves as an adhesin for intracellular interaction and attachment to abiotic surfaces during biofilm formation (Wang et al., 2004; Itoh et al., 2008). In addition to PGA, NhaR is implicated in the production of curli, which is important for attachment during biofilm formation (Kikuchi et al., 2005; Smith et al., 2017). Thus, it is likely that NhaR-suppressed cells have significantly improved biofilm formation.

There is a clear difference between YebC- and NhaR-mediated suppression, as the former alleviated the accumulation of defective LPS core, reduced capsule production, and increased biofilm formation. However, the latter did not relieve the impairment of LPS synthesis. Based on these findings, suppressors can be categorized into surface polysaccharide and ribosome suppressors. Most importantly, the suppressive NhaR modulated rRNA transcription in ESC19 at low temperature, promoting ribosome assembly, even though the strain ESC61 did not show evident ribosome defects (Supplementary Figure S8). Recently, it was reported that both the growth phase and temperature alter the activities of P*rrn* or its subpromoters, P1*rrn* and P2*rrn* (Piersimoni et al., 2016). Consistent with our results, full-length P*rrnA*, P*rrnB*, P*rrnG*, and P*rrnH* responded to low temperature with drastic transcriptional activation. At 37°C, most rRNA are transcribed from P1*rrn,* which is irresponsive to cold shock, whereas P2*rrn* of all seven *rrn* operons is the main promoter activated at low temperature. When encountering adverse environments, rRNA transcription is regulated by various trans-acting elements such as Fis, H-NS, Lrp, and DksA to optimize ribosome production (Paul et al., 2004; Hillebrand et al., 2005; Pul et al., 2005). Fis, as an activator, binds to the upstream region of P1*rrn* and facilitates the incorporation of RNA polymerase into the promoter, increasing the expression of P1*rrn*. Its action on P1*rrn* varies depending on the growth phase with differential protein abundance (60,000 copies/cell in the exponential phase and 100/cell in the stationary phase) (Ali Azam et al., 1999). The cold-inducible nucleoid-associated protein H-NS binds to P1*rrn* or its upstream region, repressing rRNA transcription, and the low activity of P1*rrn* at low temperature is suggested to be due to H-NS repression (La Teana et al., 1991). A global transcriptional regulator, Lrp, in conjunction with H-NS, blocks rRNA transcription by binding to the upstream region of P1*rrn,* exerting a synergetic effect. However, the detailed mechanism underlying the regulation of P2*rrn* at low temperature and its relevant trans-acting element(s) remain elusive. Our EMSA data revealed that NhaR could bind to both P1*rrn* and P2*rrn*. Thus, investigating whether NhaR is involved in activating P2*rrn* at low temperature is of great interest. Notably, NhaR was first identified to bind P2*rrn*.

Moreover, the *osmC* gene is induced not only by NhaR but also by the Rcs pathway, and the expression of *nhaR*, *nhaA*, and *osmC* is cold-inducible (Supplementary Figure S9) (Davalos-Garcia et al., 2001; White-Ziegler et al., 2008). This suggests that cells with membrane damage either by cold shock or by LPS defects may experience oxidative stress, producing free radicals and H_2_O_2_, thus inducing the expression of detoxifying enzymes such as OsmC. These facts suggest that BipA plays a role in oxidative stress adaptation.

In summary, the functional study of NhaR in ESC19 revealed that the novel role of NhaR may be implicated in LPS synthesis and motility, possibly acting as a negative regulator. Moreover, we found that cold-inducible NhaR might be involved in the regulation of rRNA transcription at low temperature. *Escherichia coli* cells express *bipA* at the basal level (Choi et al., 2020a), and its deletion caused the bile salt sensitivity and reduced motility even at 37°C. The *bipA* deletion along with the *nhaR* deletion resulted in more defective growth at 37°C under high salt condition. This led to the idea that BipA may be required for growth at high salt concentrations at 37°C. Therefore, further studies could shed light on BipA as a global stress-responsive regulator under various unfavorable conditions, such as salt and oxidative stress.

## Data Availability

The original contributions presented in the study are included in the article/Supplementary Material, further inquiries can be directed to the corresponding author.
