# Supplementary material for: Novel rRNA transcriptional activity of NhaR revealed by its growth recovery for the bipA-deleted Escherichia coli at low temperature
Source: Front Mol Biosci. 2023 Apr 20;10:1175889. doi: 10.3389/fmolb.2023.1175889 (PMC10157491; doi:10.3389/fmolb.2023.1175889)
Supplement: Supplementary file 1 [file DataSheet1.docx]

Supplementary Material

Novel rRNA Transcriptional Activity of NhaR Revealed by its Growth Recovery for the *bipA*-deleted *Escherichia coli* at Low Temperature

**Eunsil Choi^1,2^, Ahhyun Huh^1^, and Jihwan Hwang^1,2*^**

*** Correspondence:** Jihwan Hwang (hwangjh@pusan.ac.kr)

# Supplementary Figures and Tables

## Supplementary Figures

**
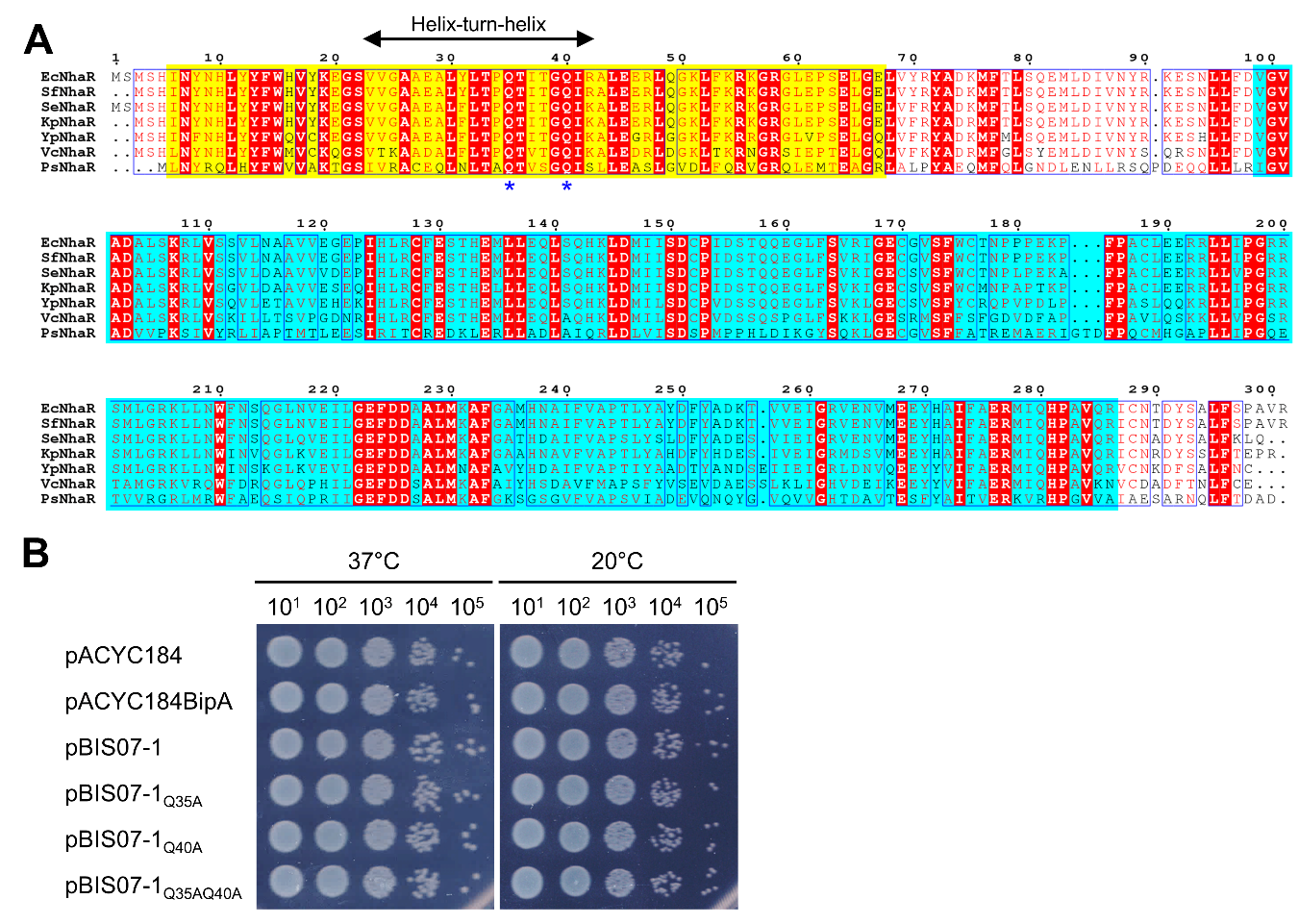
**

**Supplementary Figure S1. Effects of NhaR mutant on the growth of *E. coli* MG1655.** **(A)** The primary sequence alignment of NhaR homologs in other bacteria. The primary sequence of *E. coli* NhaR (EcNhaR: GenBank accession number, WP_000062890.1) was aligned with various NhaR homologs in *Shigella flexneri* (SfNhaR: P0A9G3.1), *Salmonella enterica* (SeNhaR: WP_079817891.1), *Klebsiella pneumoniae* (KpNhaR: WP_103527939.1), *Yersinia pestis* (YpNhaR: AJI93144.1), *Vibrio cholerae* (VcNhaR: OAE94603.1), and *Pseudomonas syringae* (PsNhaR: WP_054081492.1) using ClusterW (https://www.genome.jp/tools-bin/clustalw) and ESPript 3.0 (http://espript.ibcp.fr/ESPript/cgi-bin/ESPript.cgi). Asterisks indicate Q35 and Q40 residues at the HTH motif in the N-terminal DNA-binding domain. The N-terminal DNA-binding domain and the C-terminal co-inducer (Na^+^)-binding domain are shown in yellow and cyan, respectively. **(B)** The effect of mutant NhaR on growth of wild-type MG1655. The MG1655 transformants were spotted as carried out in **Figure 1.**


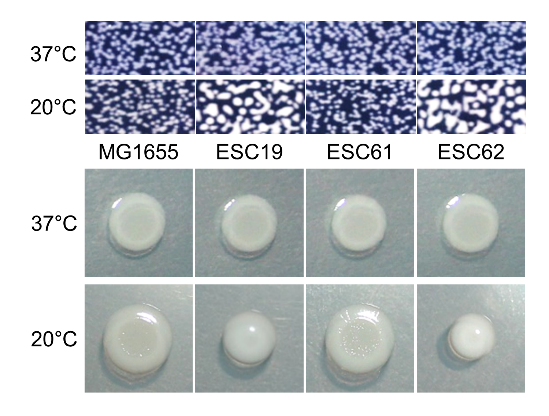


**Supplementary Figure S2. Effect of *nhaR* deletion on capsule production.** The diluted MG1655, ESC19, ESC61, and ESC62 cells were either spread (upper panel) or spotted (lower panel) on LB agar plates as carried out in **Figure 2**. The plates were incubated at 37℃ or 20℃.


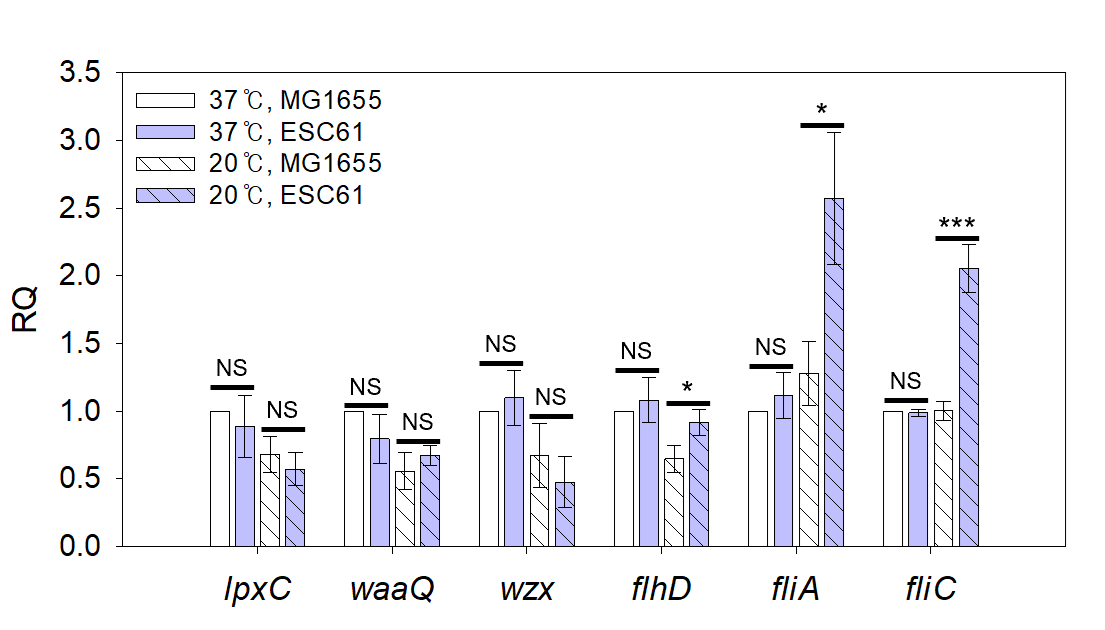


**Supplementary Figure S3. Relative quantification of mRNA levels of LPS- or flagella-related genes.** Total RNAs were extracted from MG1655 or ESC61 cells grown on LB agar plates at 37℃ or 20℃ and subjected to qRT-PCR analysis. The expression levels were normalized to *gapA* transcript. Three independent experiments were carried out and error bars denote the S.D.


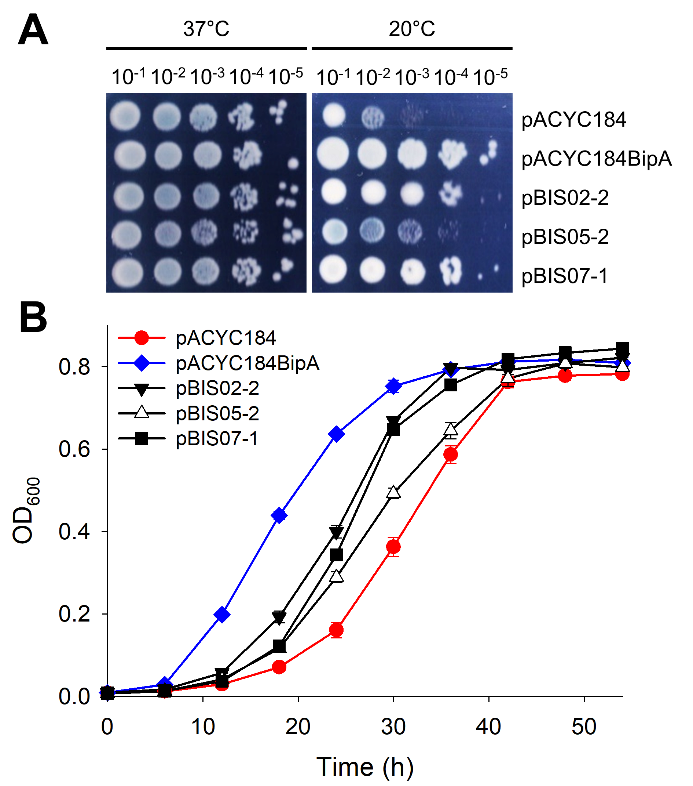


**Supplementary Figure S4. Comparison of growth suppression activities of L20, YebC, and NhaR**. **(A)** Overnight cultures of ESC19 cells harboring pACYC184, pACYC184BipA, pBIS02-2, pBIS05-2, or pBIS07-1 were diluted and spotted on LB agar plates supplemented with Kan and Cm, as described in **Figure 1**. The plates were incubated at 37℃ or 20℃. **(B)** The growth of each transformant in **(A)** was monitored as carried out in **Figure 1**. Three independent experiments were carried out and error bars represent S.D.


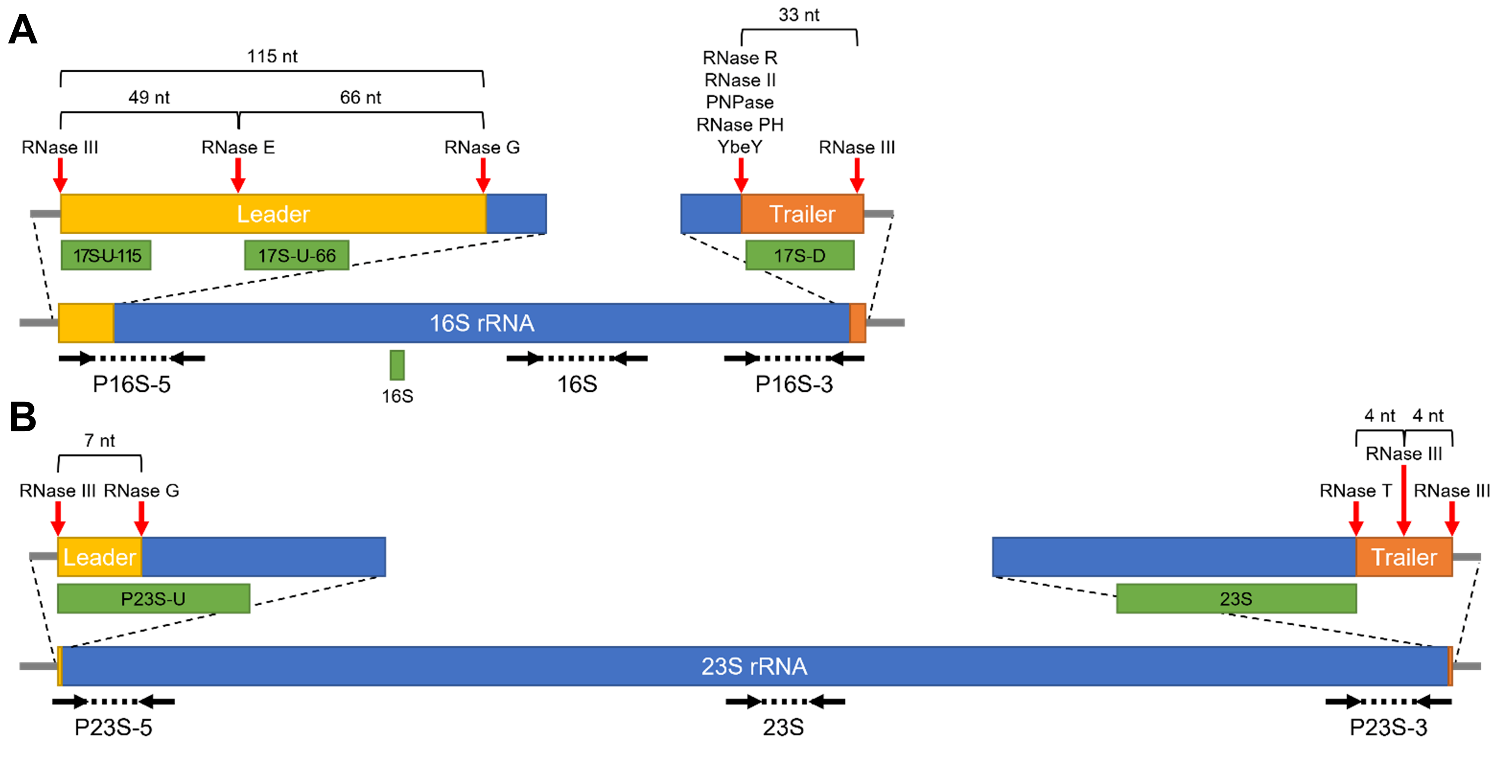


**Supplementary Figure S5. Schematic presentation of pre-16S and pre-23S rRNAs processing.** The mature 16S **(A)** and 23S **(B)** rRNAs (blue boxes) are produced by cleavage of 5ˊ leader (yellow boxes) and 3ˊ trailer (orange boxes). Cleavage sites and RNases are indicated by red arrows. The positions of biotin-labeled probes for the northern blot are shown in green boxes, and the qRT-PCR amplicons are indicated by black arrows.


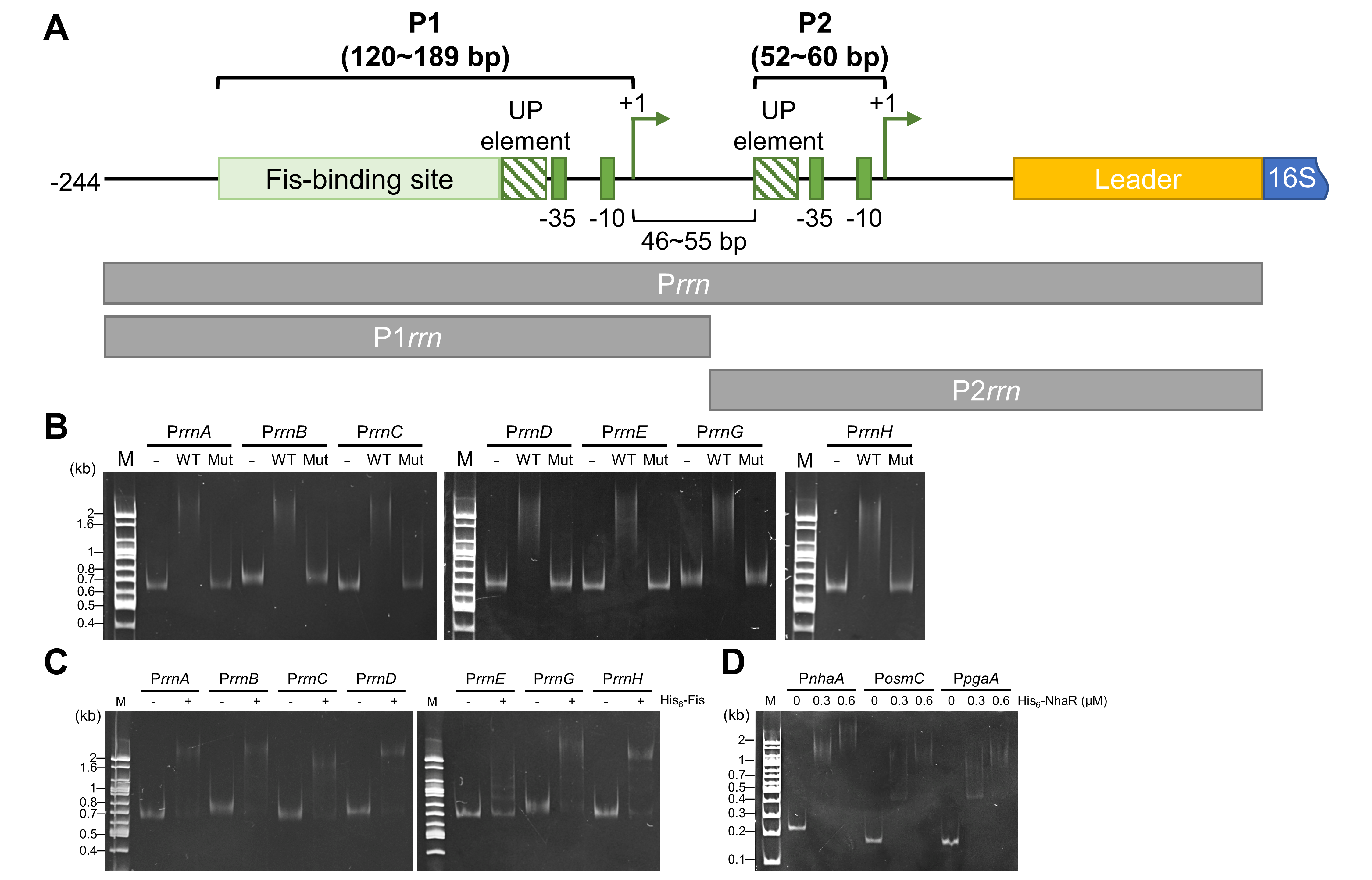


**Supplementary Figure S6. EMSA analysis. (A)** Schematic diagram of the *rrn* promoter. DNA regions corresponding to Fis-binding site, UP elements, and -35 and -10 regions are indicated as light green, green slashed, and green boxes, respectively. Nucleotides are numbered relative to the transcription start site (+1 and green arrows). **(B, C)** EMSA analysis using His_6_-NhaR_Q35AQ40A_ **(B)** or His_6_-Fis **(C)** with the promoter DNA fragments of seven *rrn* operons. 10 ng of each P*rrn* DNA fragment was incubated with 300 nM of His_6_-NhaR, mutant His_6_-NhaR_Q35AQ40A_, or His_6_-Fis. **(D)** EMSA analysis using His_6_-NhaR with the promoter DNA fragments of *nhaA*, *osmC*, or *pgaA*. 10 ng of DNA fragments were incubated with 0, 300, or 600 nM of His_6_-NhaR. M, 100-bp DNA ladder (Bioneer).

**Supplementary Figure S7. Relative quantification of the levels of 23S and 16S rRNAs.** The MG1655 cells transformed with pACYC184 or pBIS07-1 were incubated at 20℃ to the early exponential phase, after which the equal amounts of cells were harvested. The extracted RNAs were subjected to qRT-PCR analysis. RQ value is calculated using the formula: RQ = 2^-ΔCt^ (ΔCt = Ct_pACYC184_ – Ct_pBIS07-1_). Three independent experiments were carried out and error bars represent S.D.

***

**


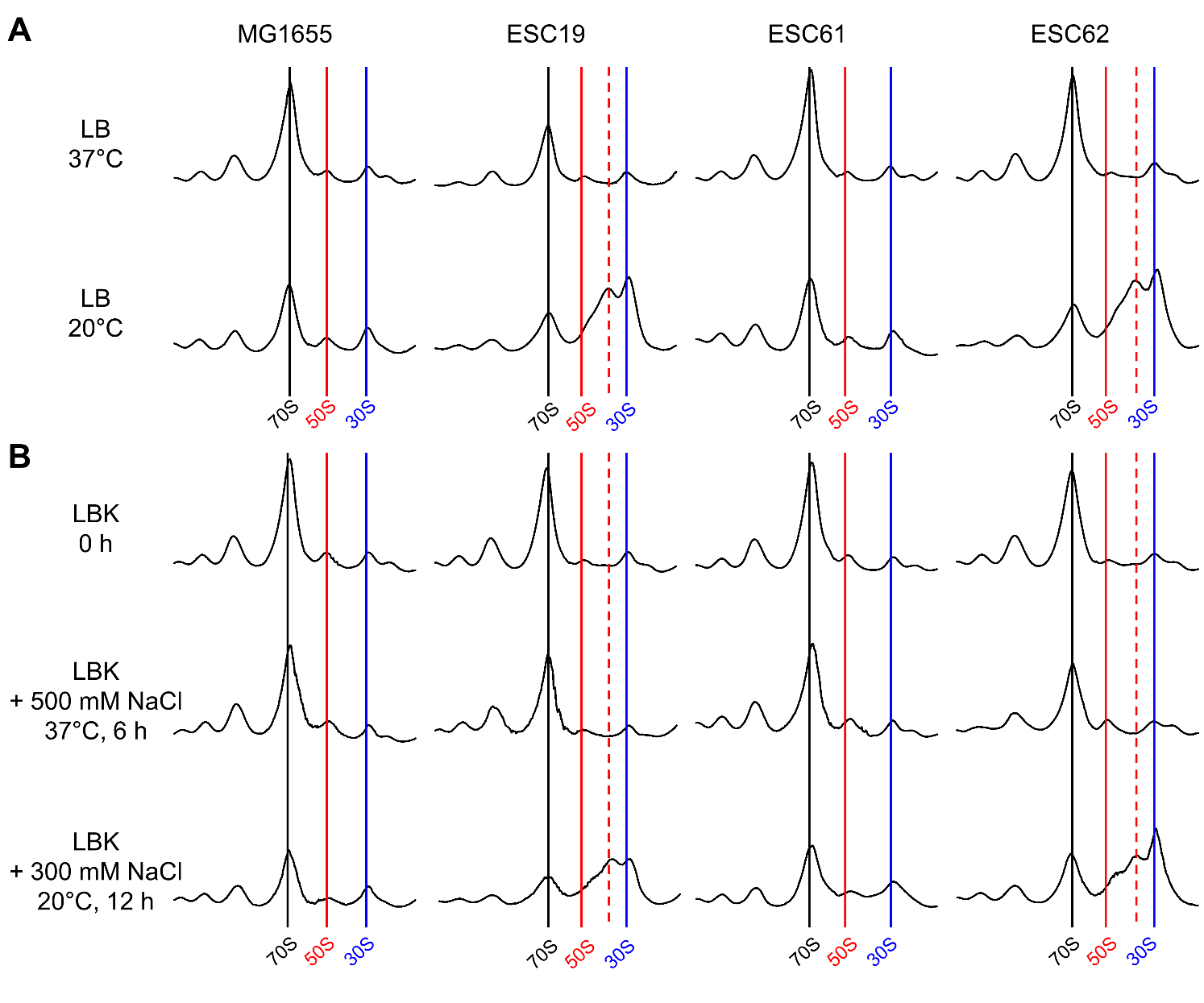


**Supplementary Figure S8. Polysome profiles of *bipA*- or *nhaR*-deleted cells.** **(A)** Polysome profiles of MG1655, ESC19, ESC61, and ESC62 cells in LB medium. Cells were grown at 37℃ or 20℃ in LB medium to the early exponential phase and harvested by centrifugation. **(B)** The effects of *bipA* or *nhaR* deletion on ribosome assembly under salt stress conditions. Cells were incubated as described in **Figure 8**. Before and after exposure to salt stress for 6 h at 37℃ or for 12 h at 20℃, cells were harvested by centrifugation and subjected to polysome profiling analysis as in **Figure 5**. Red dashed lines indicate the peak corresponding to abnormal 50S particles.


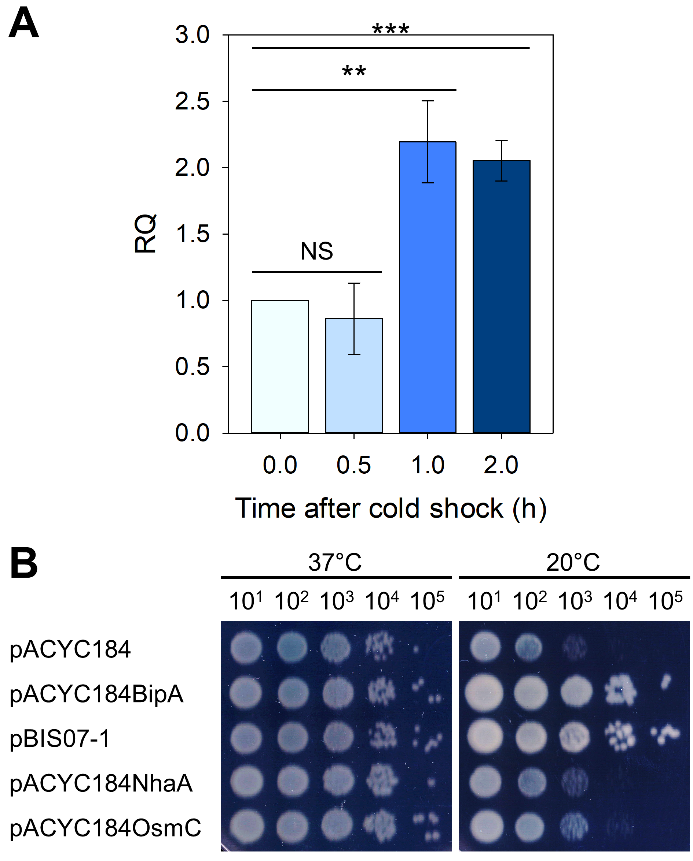


**Supplementary Figure S9. Quantification of the expression level of *nhaR* after cold shock.** The MG1655 cells were incubated at 37℃ to the early exponential phase, after which the culture was shifted to 20℃, followed by further incubation for 2 h. Cells were harvested at the indicated time points, and the extracted RNAs were subjected to qRT-PCR analysis using *rrsA* as a reference gene. Error bars represent S.D.

## Supplementary Tables

Supplementary Table S1. Primers used in this study

| Primers | Sequences (5ʼ →3ʼ) | References |
| --- | --- | --- |
| Cloning | | |
| BIS07-1-5-BamHI | AAGGATCCGATCCAGAACTGATTAACTGG | This study |
| BIS07-1-3-BamHI | AAGGATCCGTTGGAGTCATTACCAGCAA | This study |
| BIS07-2-5-BamHI | AAGGATCCTGCGTTAATCGGCAGCTC | This study |
| BIS07-2-3-BamHI | AAGGATCCATCAACAAACTGGCTTAATCGC | This study |
| nhaR-5-NdeI | ACCATATGAGCATGTCTCATATCAATTAC | This study |
| nhaR-3-SalI | TTGTCGACTTAACGCACCGCTGGACTAAAAAG | This study |
| NhaR-Q35A-F | TTTATTTAACTCCAGCAACCATTACCGGAC | This study |
| NhaR-Q35A-R | GTCCGGTAATGGTTGCTGGAGTTAAATAAAG | This study |
| NhaR-Q40A-F | AAACCATTACCGGAGCGATTCGAGCGCTG | This study |
| NhaR-Q40A-R | CAGCGCTCGAATCGCTCCGGTAATGGTTT | This study |
| NhaR-Q35AQ40A-F | CAACCATTACCGGAGCGATTCGAGCGCTG | This study |
| NhaR-Q35AQ40A-R | CAGCGCTCGAATCGCTCCGGTAATGGTTG | This study |
| fis-5-NdeI | ACCATATGTTCGAACAACGCGTAA | This study |
| fis-3-HindIII | ATAAGCTTTTAGTTCATGCCGTATTTTTTC | This study |
| PrrnA-5-EcoRI | ATGAATTCCGTTATCGCTGGTACGACCG | This study |
| PrrnA-3-BamHI | ATGGATCCGGCATGGGTAATCCTCCTGTTTAAAAGTTTGACGCTCAAAGAATTAAACTTCG | This study |
| PrrnB-5-EcoRI | ATGAATTCGATTCTGGCGCAGCGATTGC | This study |
| PrrnC-5-EcoRI | ATGAATTCGAAAGGTTTTTCTGTGCAGCTAAC | This study |
| PrrnC-3-BamHI | ATGGATCCGGCATGGGTAATCCTCCTGTTTAAAAGTTTGATGCTCAAAGAATTAAACTTCG | This study |
| PrrnD-5-EcoRI | ATGAATTCCGATCATTACGCGCTGACC | This study |
| PrrnE-5-EcoRI | ATGAATTCCTCAGCTAACGCCCCTAAC | This study |
| PrrnG-5-EcoRI | ATGAATTCGCAGCAGATCGAAAACCCG | This study |
| PrrnH-5-EcoRI | ATGAATTCGATATGCAGGCAGCGGTTG | This study |
| PrrnH-3-BamHI | ATGGATCCGGCATGGGTAATCCTCCTGTTTAAAAGTTTGATGCTCAAAGAATTAAACTTTG | This study |
| Strain construction | | |
| lacZ-150-U | CACGACAGGTTTCCCGACTG | (Choi et al., 2020b) |
| lacZ-150-D | TCGGGAAAAACGGGAAGTAGG | (Choi et al., 2020b) |
| bipA-100-U | CTCTGCAATACTTGTTTG | (Choi and Hwang, 2018) |
| bipA-100-D | CCACAGACTTATAAGGGAG | (Choi and Hwang, 2018) |
| EMSA | | |
| PnhaA-5 | CCGTCAAAAACGCATCTCACCG | This study |
| PnhaA-3 | TCCCGAGGCATCACTGCTAAAG | This study |
| PosmC-5 | CCCGGTAATCTATTGTGGG | This study |
| PosmC-3 | GTTGCTCTCCTGTGGGC | This study |
| PpgaA-5 | CAATTAAATCCGTGAGTGCCG | This study |
| PpgaA-3 | TCTTCAGGAATACGGCATAAAT | This study |
| rrnABCG-P1-3 | CGGCGTGTTTGCCGTTGTTC | This study |
| rrnD-P1-3 | GTTCCGACCATCCTGTGAAGTG | This study |
| rrnEH-P1-3 | CGAACCGGCTGTTTGTGTGAAG | This study |
| rrnABC-P2-5 | CCGGGTCAGCGGGGTTC | This study |
| rrnG-P2-5 | CCGGGTCGGCGGGGTTC | This study |
| rrnEH-P2-5 | GTTCGGTTGAAGAGAAAAATCCTG | This study |
| BCBS-II-F | AATGACCTTTGGATAACCCTTTTTATGCTCCGTGAAAGCGATCACAAAGGGACTCTGCAATACTTGTTTGCG | (Choi and Hwang, 2018) |
| BCBS-II-R | CGCAAACAAGTATTGCAGAGTCCCTTTGTGATCGCTTTCACGGAGCATAAAAAGGGTTATCCAAAGGTCATT | (Choi and Hwang, 2018) |
| qRT-PCR | | |
| rrlA-RT-F | ATATTCCTGTACTTGGTGTT | (Choi et al., 2020b) |
| rrlA-RT-R | CTTGGTATTCTCTACCTGAC | (Choi et al., 2020b) |
| rrsA-RT-F | GACTTGGAGGTTGTGCCCTT | (Choi et al., 2020b) |
| rrsA-RT-R | GATAAGGGTTGCGCTCGTTG | (Choi et al., 2020b) |
| P23S-5F | ATCTTCGGGTTGTGAGG | (Choi et al., 2020b) |
| P23S-5R | GGAATCTCGGTTGATTTC | (Choi et al., 2020b) |
| P23S-3F | CTAGTACGAGAGGACCGG | (Choi et al., 2020b) |
| P23S-3R | CGGCGTTGTAAGGTTAAG | (Choi et al., 2020b) |
| P16S-5F | GTGGGCACTCGAAGATACGG | (Choi et al., 2020b) |
| P16S-5R | TCTTGCGACGTTATGCGGT | (Choi et al., 2020b) |
| P16S-3F | GAGAGCAAGCGGACCTCATA | (Choi et al., 2020b) |
| P16S-3R | TGTGAGCACTTCAAAGAACGC | (Choi et al., 2020b) |
| nhaA-RT-5 | TGGCGGTATTTTTCCTGTTA | This study |
| nhaA-RT-3 | ATAGCCAGAGCCATCAAAAA | This study |
| nhaR-RT-5 | TACAAATCCACCACCAGAAA | This study |
| nhaR-RT-3 | TCTACGACAGTTTTATCGGC | This study |
| osmC-RT-5 | AACAGTATCCACCGAGAGT | This study |
| osmC-RT-3 | ACTTTATCCAGCGACACATC | This study |
| lpxC-RT-5 | ATGGTTTTTCGCTGGATTTC | (Choi et al., 2020a) |
| lpxC-RT-3 | CTTCGTTCAGTACGCGATAA | (Choi et al., 2020a) |
| waaQ-RT-5 | ATTTCTACACCGACACAACA | (Choi et al., 2020a) |
| waaQ-RT-3 | ATCTCATCTACACAAGCGAG | (Choi et al., 2020a) |
| wzx-RT-5 | TTCACTTGTACGGTGGTTAG | (Choi et al., 2020a) |
| wzx-RT-3 | ATAGTTGATGATGCTGTCCG | (Choi et al., 2020a) |
| flhD-RT-5 | TCTTGCGTCAACTGAGTAAT | This study |
| flhD-RT-3 | CGAGTTGCTGAAACACATT | This study |
| fliA-RT-5 | GATTCAGCTCTTCCTGGTAA | This study |
| fliA-RT-3 | ATTATCGCCAAATGTTGCTC | This study |
| fliC-RT-5 | CGCCTGAAGTGATAGTTGTA | This study |
| fliC-RT-3 | ATTACCCTTTCTACGGAAGC | This study |
| gapA-RT-5 | AAGTTGGTGTTGACGTTGTCGC | (Choi and Hwang, 2018) |
| gapA-RT-3 | AGCGCCTTTAACGAACATCG | (Choi and Hwang, 2018) |
| Northern blotting | | |
| 23S-M | AAGGTTAAGCCTCACGGTTC | (Choi et al., 2020b) |
| P23S-U | CGCTTAACCTCACAAC | (Choi et al., 2020b) |
| 16S-M | CATCTGACTTAACAAACCGCCTGCGT | This study |
| P16S-U | GAATTACGTGTTCACTCTTGAGACTTGG | This study |
| P16S-D | GTGTGAGCACTTCAAAGAACGCTTCTTTAAG | This study |

# References

Choi, E., and Hwang, J. (2018). The GTPase BipA expressed at low temperature in *Escherichia coli* assists ribosome assembly and has chaperone-like activity. *J Biol Chem* 293, 18404-18419.

Choi, E., Jeon, H., Oh, C., and Hwang, J. (2020a). Elucidation of a Novel Role of YebC in Surface Polysaccharides Regulation of *Escherichia coli bipA*-Deletion. *Front Microbiol* 11, 597515.

Choi, E., Jeon, H., Oh, J.I., and Hwang, J. (2020b). Overexpressed L20 Rescues 50S Ribosomal Subunit Assembly Defects of *bipA*-deletion in *Escherichia coli*. *Front Microbiol* 10, 2982.
